# H3K27M Mutation Modulates the Antigenic Landscape in Diffuse Midline Glioma

**DOI:** 10.3390/cancers18193142

**Published:** 2026-09-28

**Authors:** Tima Shamekhi, Bijun Zeng, Claire Xin Sun, Paul Daniel, Terry C. C. Lim Kam Sian, Gabriel Goncalves, Grace Huang, Farnaz Fahimi, Nivedhitha Selvakumar, Erwin Tanuwidjaya, Isaac Woodhouse, Bettina Kritzer, Ralf B. Schittenhelm, Roberta Mazzieri, Jason E. Cain, Javad Nazarian, Jordan R. Hansford, Ron Firestein, Riccardo Dolcetti, Pouya Faridi

**Affiliations:** 1 Centre for Cancer Research, Hudson Institute of Medical Research, Clayton, VIC 3168, Australia; tima.shamekhi@monash.edu (T.S.); claire.sun@hudson.org.au (C.X.S.); paul.daniel@hudson.org.au (P.D.); terry.lim@monash.edu (T.C.C.L.K.S.); gabriel.goncalves@monash.edu (G.G.); grace.huang@monash.edu (G.H.); farnaz.fahimi@monash.edu (F.F.); nivedhitha.selvakumar@monash.edu (N.S.); erwin.tanuwidjaya1@monash.edu (E.T.); isaac.woodhouse@monash.edu (I.W.); jason.cain@hudson.org.au (J.E.C.); ron.firestein@hudson.org.au (R.F.); 2 Department of Medicine, School of Clinical Sciences, Monash University, Clayton, VIC 3168, Australia; 3 Peter MacCallum Cancer Centre, Melbourne, VIC 3000, Australia; bijun.zeng@petermac.org (B.Z.); roberta.mazzieri@petermac.org (R.M.); 4 Sir Peter MacCallum Department of Oncology, The University of Melbourne, Parkville, VIC 3010, Australia; 5 Monash Proteomics and Metabolomics Platform, Biomedicine Discovery Institute, Monash University, Clayton, VIC 3800, Australia; ralf.schittenhelm@monash.edu; 6 Department of Paediatrics, University Children’s Hospital Zurich, 8008 Zurich, Switzerland; bettina.kritzer@kispi.uzh.ch (B.K.); javad.nazarian@uzh.ch (J.N.); 7 Department of Molecular and Translational Science, School of Medicine, Nursing and Health Sciences, Monash University, Clayton, VIC 3800, Australia; 8 Department of Paediatrics, Monash University, Clayton, VIC 3168, Australia; 9 Michael Rice Centre for Hematology and Oncology, Women’s and Children’s Hospital, South Australia Health and Medical Research Institute, South Australia Immunogenomics Cancer Institute, Adelaide University, Ade-laide, SA 5000, Australia; jordan.hansford@sa.gov.au; 10 Department of Microbiology and Immunology, The University of Melbourne, Parkville, VIC 3010, Australia

**Keywords:** immunopeptidomics, antigen processing and presentation, paediatric diffuse midline glioma, H3K27M, tumour-associated antigens, PRAME, T cell immunotherapy

## Abstract

Background/Objectives: Diffuse midline gliomas (DMGs) are among the most aggressive paediatric brain tumours, with the pathognomonic H3K27M mutation present in over 80% of cases. This mutation drives epigenetic dysregulation and transcriptional reprogramming, yet its impact on the tumour antigenic landscape remains poorly understood. Given the low mutational burden of DMG, an expanded search beyond neoantigens to include epigenetically dysregulated tumour-associated antigens (TAAs) is critical for advancing antigen-specific immunotherapies. Methods: We performed immunopeptidomic analysis of two patient-derived H3K27M-mutant DMG cell lines (SU-DIPG13 and BT245) and their isogenic counterparts in which only the mutant H3F3A allele was disrupted by CRISPR editing (H3K27M-KO), leaving wild-type histone H3.3 intact. High-resolution mass spectrometry and bioinformatics were used to define H3K27M-associated changes in the immunopeptidome, and functional T cell assays with HLA-matched healthy donor PBMCs assessed peptide immunogenicity. Results: H3K27M modulated the tumour antigenic landscape in a model-specific manner. Loss of the mutant allele increased HLA-I expression in SU-DIPG13 but not BT245, while immunopeptidomic profiling revealed distinct shifts in tumour-associated peptide presentation in both models, indicating that HLA-I abundance alone does not explain these changes. We identified six peptides derived from SLITRK2, PRAME, XKR5 and CBX2 that elicited T-cell responses in vitro; T cells raised against five of these preferentially recognised the parental H3K27M-mutant DMG cells. Several prioritised peptides were detected only in parental H3K27M-mutant cells, supporting mutation-associated, antigen-selective differences in peptide presentation and tumour antigenicity. Conclusions: This study shows that H3K27M modulates the antigenic landscape in DMG in a model-dependent, antigen-selective manner, uncovering immunogenic tumour-associated peptides that could serve as targets for precision immunotherapy and establishing a foundation for T cell-based therapies in this otherwise treatment-refractory disease.

## Simple Summary

Diffuse midline glioma is an aggressive brain tumour of childhood for which there is no curative treatment. Almost all these tumours carry a single change in a histone protein, called H3K27M, which alters how genes are switched on and off. T cells can kill tumour cells, but only if they can see fragments of tumour proteins displayed on the tumour cell surface. We asked whether the H3K27M change alters which fragments are displayed. Comparing tumour cells carrying the change with matched cells in which it had been removed, we found that the change shifts the set of displayed fragments. Six fragments elicited responses from healthy-donor T cells, and T cells raised against five preferentially recognised tumour cells carrying the change. These fragments are candidate targets for T-cell-based treatments, and our approach shows how tumours driven by such changes can be made visible to the immune system.

## 1. Introduction

Diffuse midline gliomas (DMGs) represent one of the most aggressive and lethal forms of paediatric brain tumours, with a median survival of 9–11 months [1,2]. The heterozygous somatic histone mutation H3K27M is present in over 80% of DMG cases, especially in diffuse intrinsic pontine gliomas (DIPGs) [3,4]. This mutation affects key histone genes: HIST1H3B/C, which encode the canonical histone H3.1, and H3F3A, encoding the variant histone H3.3. H3.3 accounts for roughly 70% of H3K27M mutations and is correlated with a particularly poor prognosis [5,6].

Mechanistically, substituting lysine 27 with methionine in histone H3 triggers widespread epigenetic reprogramming. This mutation results in a global loss of H3K27 di- and tri-methylation, increased acetylation, and alterations in three-dimensional chromatin architecture, collectively leading to the dysregulation of DNA methylation and gene expression [7,8]. Although only 3–17% of total cellular histone H3 proteins are mutated, the resulting dominant hypomethylation disrupts transcriptional silencing and promotes the upregulation of proto-oncogenes while repressing genes involved in cellular differentiation [9,10]. Beyond driving oncogenesis, these epigenetic alterations induce a stem cell-like, immune-cold tumour state and modulate antigen presentation and T cell infiltration, thereby facilitating immune evasion [9,11,12,13,14,15,16].

The advent of immunotherapy, particularly adoptive T cell-based approaches, offers a promising strategy to overcome the inherent resistance of DMG to conventional therapies by enhancing tumour immunogenicity. Effective anti-tumour immunity is largely mediated by CD8^+^ T cells that recognise tumour antigens presented by major histocompatibility complex (MHC) class I molecules (known as human leukocyte antigens, HLA, in humans). Typically, these antigens are peptide fragments derived from tumour-specific mutations or dysregulated proteins [17,18]. Peptide antigens derived from the H3K27M mutation represent promising targets for peptide–HLA (pHLA)-mediated immunotherapy due to their tumour specificity. However, previous efforts to identify HLA-A*02:01–restricted peptide neoantigens derived from this mutant protein have yielded inconclusive results, underscoring the need to expand the search for other actionable cancer antigens [19,20,21].

The immune-privileged nature of the brain poses additional challenges for effective immunotherapy in DMG [22]. Overcoming these barriers requires a comprehensive understanding of the tumour immunopeptidome to drive the development of targeted therapeutic strategies. To bridge this gap, we performed an in-depth immunopeptidomic analysis to systematically define the landscape of tumour-associated antigens in DMG. Given the inherently low mutational burden of DMG and the challenge of identifying H3K27M-derived neoantigens [9,20,23], we expanded our search to include non-mutated but aberrantly expressed antigens influenced by the tumour’s epigenetic dysregulation. Using state-of-the-art mass spectrometry and robust bioinformatic pipelines, we sought to determine how the H3K27M mutation modulates antigen presentation and whether it gives rise to immunogenic pHLA complexes. To achieve this, we leveraged two patient-derived H3K27M-mutant DMG cell line models, SU-DIPG13 (derived from a pontine diffuse intrinsic pontine glioma) and BT245 (derived from a thalamic high-grade glioma), and their CRISPR-edited H3K27M mutant-allele knockout (H3K27M-KO) counterparts [7]. Importantly, H3K27M arises as a heterozygous somatic mutation, and CRISPR editing in these models disrupted only the single mutant H3F3A allele: the wild-type H3F3A allele and all other histone H3 genes remain intact, so the KO lines continue to express wild-type histone H3.3. These lines are therefore mutant-allele knockouts rather than histone H3 knockouts. They therefore represent the closest attainable isogenic comparator lacking the mutant H3K27M allele for this disease, since H3K27M is present in the founding tumour clone and no patient-matched H3K27M-negative DMG line exists, while knock-in reversion of the mutant residue to wild-type lysine is not feasible in these models. Comparing H3K27M-mutant cells with their mutant-allele-knockout derivatives thus isolates the contribution of the mutant allele on an otherwise identical genetic background. These models provide a controlled isogenic background, minimising confounding variables and enabling a direct evaluation of the mutation’s impact. Accordingly, this study was designed to assess H3K27M-associated changes in HLA-I peptide presentation using controlled isogenic models, rather than to define the broader DMG immunopeptidome. Moreover, we functionally assessed the immunogenicity of the identified pHLA complexes to pinpoint novel actionable targets for T cell-mediated immunotherapy.

By integrating these advanced methodologies, our study provides a comprehensive analysis of H3K27M-mutant DMG, offering crucial insights into how this mutation modulates the tumour’s antigenic landscape. We identify antigenic changes associated with H3K27M-driven epigenetic dysregulation and the generation of targetable antigens. This work not only enhances our understanding of the immune landscape in DMG but also lays the foundation for novel, personalised immunotherapeutic strategies aimed at overcoming the immune-evasive nature of these highly aggressive tumours.

## 2. Materials and Methods

The experimental procedures were designed to investigate the immunogenic profile of H3K27M-mutant cells by analysing the altered immunopeptidome and antigen presentation using isogenic models of paediatric diffuse midline glioma (pDMG).

### 2.1. Cell Culture

Two patient-derived H3K27M-isogenic cell line pairs (parental H3K27M-mutant and H3K27M-KO), SU-DIPG13 and BT245, were used in this study. The parental H3K27M-mutant cell lines were originally established at Stanford University (SU-DIPG13) and Boston Children’s Hospital (BT245). Subsequently, the H3K27M-KO variants were generated by Dr. Nada Jabado’s team at McGill University [7]. In these models CRISPR-Cas9 editing was allele-specific: only the single mutant copy of H3F3A carrying the K27M substitution was disrupted, while the remaining wild-type H3F3A allele and all other canonical and non-canonical histone H3 genes were left unmodified. The edited lines therefore retain expression of wild-type histone H3.3 and differ from their parental counterparts solely by loss of the H3K27M-mutant allele. For clarity, these lines are referred to throughout as H3K27M-KO, denoting knockout of the mutant allele, and should not be interpreted as histone H3 knockouts. Cells were maintained in tumour stem medium (TSM). Tumour-base medium (TBM) was prepared by mixing Neurobasal-A and Dulbecco’s Modified Eagle Medium/F-12 (DMEM/F-12) (1:1 ratio) (Gibco, Thermo Fisher Scientific, Waltham, MA, USA), supplemented with 10 mM HEPES, 1 mM sodium pyruvate, 1× MEM non-essential amino acids, 1× GlutaMAX, and 1% penicillin–streptomycin (Gibco). TSM was prepared by supplementing TBM with 1× B27 supplement (minus vitamin A), 20 ng/mL EGF, 20 ng/mL FGF-basic, 10 ng/mL PDGF-AA, 10 ng/mL PDGF-BB, and 2 µg/mL heparin (STEMCELL Technologies, Vancouver, BC, Canada). Cells were detached using Accutase (Sigma-Aldrich, St. Louis, MO, USA), washed with 1× PBS, and subsequently pelleted by centrifugation at 2500× *g* for 15 min at 4 °C. Cell pellets were snap-frozen in liquid nitrogen and stored at −80 °C until further analysis.

### 2.2. High-Resolution HLA Typing

Total DNA was extracted from 5 × 10^6^ cells using the DNeasy Blood & Tissue Kit (Qiagen, Hilden, Germany) according to the manufacturer’s instructions. Samples were then sent to the Australian Red Cross Lifeblood for Next-Generation Sequencing (NGS)-based profiling of HLA class I.

### 2.3. RNA Sequencing

We used previously published RNA-seq data [24]. Briefly, RNA was extracted with Qiagen RNeasy columns according to the manufacturer’s instructions, including on-column DNase treatment. All RNA-seq samples were submitted for sequencing in triplicate. Library preparation and RNA-seq were performed at Beijing Genomics Institute with a minimum of 20 million reads per sample using paired-end 150-bp sequencing. Ribosomal RNA was depleted and poly(A) RNA was enriched from the total RNA samples. The library was then sequenced on a DNBSEQ G-400 platform. Data processing was automated using the RNAsik pipeline. Differential expression analysis was performed using the Degust platform with the limma–voom method [25]. A permissive threshold was applied to determine significant differential expression of genes in the H3K27M knockout (KO) groups compared to the parental (H3K27M-mutant) lines, defined as |log2 fold change| > 1 with an adjusted *p* value < 0.05.

### 2.4. Flow Cytometry

Cells were cultured under basal conditions or treated with 5 ng/mL interferon-gamma (IFN-γ) (Sino Biological, Beijing, China) for 48 h and then stained with a pan-HLA class I antibody (W6/32) conjugated to FITC (Leinco Technologies, St. Louis, MO, USA). Fluorescence intensity was assessed by flow cytometry to evaluate changes in cell surface HLA-I expression. All flow cytometric assays were acquired using a BD LSRFortessa™ cell analyser (BD Biosciences, San Jose, CA, USA) at the FlowCore facility, Monash University. Data were analysed using FlowJo^®^ software version 10.10.0 (Becton, Dickinson and Company, Ashland, OR, USA). Statistical analyses were performed using GraphPad Prism, version 10.4.1.

### 2.5. Purification of HLA–Peptide Complexes from Cell Pellets

HLA–peptide complexes were isolated using the semi-automated SAPrIm method on the KingFisher platform, as previously described [26]. Briefly, cell pellets were lysed in 500 µL of lysis buffer containing 1% CHAPS (Thermo Fisher Scientific, Waltham, MA, USA), 50 mM Tris (pH 8.0), 150 mM NaCl, 10 mM 2-chloroacetamide (CAA; for cysteine alkylation) (Sigma-Aldrich, St. Louis, MO, USA), and 1× Halt™ Protease and Phosphatase Inhibitor Single-Use Cocktail (Thermo Fisher Scientific). Lysates were gently mixed and incubated on a roller at 4 °C for 1 h.

HLA–peptide complexes were immunoprecipitated using hyper-porous magnetic protein A beads (MagReSyn, ReSyn Biosciences, Pretoria, South Africa) conjugated with the pan anti-HLA-I antibody W6/32 (Leinco Technologies). Peptide ligands were eluted under acidic conditions and subsequently desalted and concentrated using C18 resin columns (BioPureSPN, The Nest Group, Ipswich, MA, USA) to remove buffer components, detergents, and salts that could interfere with downstream LC-MS/MS analysis.

To enhance the depth of peptide identification by LC-MS/MS, peptides were fractionated using stepwise elution with acetonitrile (ACN) at 5, 10, 15, 20, 25, and 28%. To maximise the recovery of distinct peptide species while maintaining sample complexity for optimal MS analysis, fractions were pooled as follows: 5% and 20% (Pool 1), 10% and 25% (Pool 2), and 15% and 28% (Pool 3). Fractions were then dried under a vacuum concentrator (Labconco Corporation, Kansas City, MO, USA) and stored at −80 °C until LC-MS/MS analysis to preserve sample integrity and prevent degradation.

### 2.6. LC-MS/MS Data Acquisition

Peptides were analysed using a Data-Dependent Acquisition (DDA) approach. Prior to LC-MS/MS, peptides were reconstituted in 2% acetonitrile (ACN)/0.1% trifluoroacetic acid (TFA) and loaded onto a trap-and-elute system consisting of a Thermo Fisher Scientific (Waltham, MA, USA) Acclaim™ PepMap™ 100 nanoViper C18 trap column (50 mm × 300 µm, 5 µm, 100 Å) and an Acclaim™ PepMap™ 100 C18 analytical column (50 cm × 75 µm, 2 µm, 100 Å) on an Orbitrap Exploris 480 mass spectrometer coupled to an UltiMate 3000 RSLCnano UHPLC system. Peptides were separated using a 90-min linear gradient from 6% to 36% buffer B (80% ACN/0.1% formic acid), followed by an increase to 99% buffer B at 103 min, held for 5 min, at a flow rate of 250 nL/min. MS1 spectra were acquired in the *m*/*z* range of 350–1700 at a resolution of 120,000 with an RF lens setting of 40%. Monoisotopic peak determination was set to Peptide with relaxed restriction. The automatic gain control (AGC) target was set to 250% or until reaching a maximum injection time of 50 ms. Precursor ions with charge states +1 to +4 were selected, and dynamic exclusion was applied for 10 s within a 15-ppm mass tolerance. For MS2 scans, precursor ions were isolated using a 1.1 *m*/*z* window and fragmented by higher-energy collisional dissociation (HCD) at a normalized collision energy of 30%. Fragment ions were detected at a resolution of 15,000 with a scan range mode set to ‘define first mass’ at 110 *m*/*z*. The AGC target was set to 200% or until reaching a maximum injection time of 100 ms.

### 2.7. HLA–Peptide Identification

MS/MS spectra were analysed using PEAKS 11 software (Bioinformatics Solution Inc., Waterloo, ON, Canada). Raw MS files were imported with a parent mass error tolerance of 15 ppm and a fragment mass error tolerance of 0.02 Da. Protein grouping was disabled to allow all potential protein sources for a given peptide sequence to be annotated.

An initial de novo sequencing of MS/MS spectra was performed, followed by a database search against the human proteome. The search parameters included the following variable post-translational modifications (PTMs): oxidation of methionine (M), acetylation of lysine (K), carbamidomethylation of cysteine (C), cysteinylation of cysteine (C), deamidation (N/Q), and methylation (K/R). A false discovery rate (FDR) threshold of 1% was applied at the peptide level, and all confidently identified peptides were exported for further analysis. The search database comprised the reviewed human UniProt/SwissProt proteome (~20,400 sequences), with a common contaminants database appended. Searches were performed with no enzyme specificity. No isotopic labelling or label-free quantification was applied: samples were compared on the basis of peptide identification only, and no abundance normalisation was performed. Peptide length was restricted to 8–15 residues, consistent with the expected size range of HLA class I ligands, and a maximum of three variable modifications was permitted per peptide.

### 2.8. Peptide Binding Prediction and Peptide–HLA Binding Modelling

Peptide binding predictions were performed using NetMHCpan 4.1 [27], a tool based on artificial neural networks that predicts peptide binding to HLA molecules. Peptides with a predicted percentile rank ≤ 2% were classified as binders; all others were considered unassigned. AlphaFold3 was used to predict peptide–HLA complex structures, and PyMOL, version 4.6.0 (Schrödinger, LLC, New York, NY, USA) was used for supportive assessments of HLA assignment and structural plausibility [28].

### 2.9. Criteria for Selection of Peptides for Immunogenicity Assays

To qualify as pHLA antigens, peptides were required to map to a single source protein and be absent from a published benign-tissue immunopeptidomics atlas [29]. As a final quality control step, only peptides detected in parental cells and not detected in the matched H3K27M-KO cells, derived from TAAs and supported by high-confidence MS2 spectra were selected for synthesis and spectral validation prior to immunogenicity assays. The TAA database, comprising 129 entries, was curated in-house based on a structured literature review, completed in March 2024, of genes reported as tumour-associated antigens or as dysregulated in gliomagenesis and oncogenic signalling (Supplementary Table S1). Genes were included if they met at least one of five criteria: annotation as a cancer/testis antigen; prior report as a tumour antigen in any malignancy; reported association with glioma or other central nervous system tumours; a role in oncogenic signalling pathways dysregulated in DMG; or derivation from an endogenous retroviral element. The basis for inclusion of each of the 129 entries is recorded in Supplementary Table S1. The TAA panel was applied as a predefined downstream prioritisation criterion after global peptide identification and was not used to restrict the initial MS database search or define the overall immunopeptidome. Data sources included The Cancer Genome Atlas (TCGA), the cancer proteome in the Human Protein Atlas (HPA) [30] and Childhood Cancer Model Atlas (CCMA) [24]. The expression of gene sources for the selected peptides was screened on tissue samples to ensure adequate presentation levels in actual tumour tissue. For RNA isolation, fresh-frozen DMG brainstem tumour tissue samples (5 mg) from 39 patients were processed. RNA was extracted using the RNeasy Plus Micro Kit (Qiagen, 74034). NanoDrop and Qubit were used for RNA concentration measurement and a minimum of 200 ng RNA was used for the library preparation (RNA-seq—TruSeq mRNA-seq library). RNA sequencing was performed on an Illumina (San Diego, CA, USA) NovaSeq 6000 using 50-bp paired-end reads to a depth of 25 million clusters per library. RNA-seq data were obtained as FASTQ files from all samples and processed using the nf-core/rnaseq pipeline (v 3.14.0) with default parameters [31]. Gene expression quantification was performed using Salmon (version 1.4.0), and the resulting count matrix was normalised using the DESeq2 package (v. 1.32.0) for analysis and visualisation of gene expression across the tumour samples [32,33].

### 2.10. Immunogenicity Assay of Selected Peptides

Fresh donor peripheral blood mononuclear cells (PBMCs) were isolated from buffy coats using Ficoll-Paque density centrifugation and cryopreserved in 90% foetal calf serum (FCS) and 10% dimethyl sulfoxide (DMSO) and stored in liquid nitrogen. For each peptide, PBMCs from three independent HLA-matched healthy donors were used. For antigen-specific T cell expansion, 0.25 × 10^6^ PBMC per well were cultivated in 200 µL of AIM V medium in 96-well round bottomed plates in the presence of 1 µg/mL peptide. In specific experiments, unrelated immunogenic peptides (Flu NP HLA-A*01:01) were also used to generate specific T cell cultures as a control of T cell functionality. Recombinant human interleukin (IL)-2, IL-7 and IL-15 were added one day after. A quarter of cell culture media in each well was replaced with fresh AIM V media supplemented with IL-2, IL-7 and IL-15 every second day. Cultures were harvested on day 14 for functional assay. Specific cytotoxic activity of cultured T cells against autologous PHA T blasts un-pulsed or pulsed with relevant peptide was measured by standard 51Cr release assay [34]. The percentage of specific lysis was calculated using the following formula:

(Chromium release for condition of interest − Chromium release in spontaneous release wells)/(maximum chromium release − Chromium release in spontaneous wells) × 100.

The same cytotoxicity assay was used to investigate the ability of peptide-specific T cells to lyse the parental and the H3K27M-KO cell lines. The natural killer cell-sensitive K562 cell line was also used as a target to assess the level of non-specific killing.

### 2.11. Experimental Design and Statistical Rationale

This study used a paired isogenic design. Two independent patient-derived H3K27M-mutant DMG cell lines (SU-DIPG13 and BT245) and their matched mutant-allele-knockout derivatives were analysed under two conditions, basal and IFN-γ-stimulated, giving eight sample groups. Two biologically independent models were included so that H3K27M-dependent effects could be distinguished from line-specific effects, and the paired isogenic design controls for HLA genotype, culture conditions and genetic background, allowing each KO line to serve as the internal control for its own parental line.

For the immunopeptidomic analyses, one biological sample (an independent cell pellet of approximately 5 × 10^7^ cells) was processed per cell line and per condition, and each sample was fractionated by stepwise acetonitrile elution and pooled into three fractions, each injected once, giving three LC-MS/MS acquisitions per sample. Replication in this study is therefore provided at the level of independent biological models rather than by repeated sampling of the same model: the primary comparison is between two independently derived, isogenically paired cell line systems, and only findings concordant across both pairs, or subsequently confirmed by functional T cell assays, are presented as conclusions. We note this as a constraint of the study, as the absence of biological replicates within each cell line precludes statistical testing of differential peptide presentation.

Statistical analyses were performed in GraphPad Prism. Flow cytometry data are presented as mean fluorescence intensity from three independent experiments, and comparisons between parental and KO lines, and between untreated and IFN-γ-treated cells, were made using unpaired two-tailed Student’s *t*-tests. Cytotoxicity assays, which compare target conditions across a range of effector:target ratios, were analysed by two-way ANOVA with correction for multiple comparisons. Differential gene expression in the cell line RNA-seq data was assessed with limma–voom, applying thresholds of |log2 fold change| > 1 and adjusted *p* < 0.05, with the Benjamini–Hochberg procedure used to control the false discovery rate. For the patient tissue RNA-seq dataset, DESeq2 was used for count normalisation and expression analysis; no differential-expression contrast was performed. Immunopeptidomic comparisons are qualitative: peptides were compared on the basis of identification at a 1% peptide-level FDR, and no statistical testing of peptide abundance was performed.

## 3. Results

### 3.1. H3K27M Mutation Induces Global Transcriptional Reprogramming

To elucidate the global transcriptional changes associated with H3K27M mutation, we investigated the gene expression profiles of the models. In total, 16,667 and 16,884 protein-coding genes were detected as transcribed in BT245 and SU-DIPG13 pairs, respectively (Supplementary Table S2). Following allele-specific knockout of the mutant H3F3A copy encoding K27M, 19.39% of detected genes in BT245 and 16.58% in SU-DIPG13 showed a significant twofold or greater shift in expression levels (Figure 1A). Amongst the differentially expressed genes, 622 showed a significant downregulation in both models following H3K27M-KO (Figure 1B). Gene ontology enrichment analysis of these 622 genes using Metascape [35] revealed their association with neuronal processes, including trans-synaptic signalling, modulation of chemical synaptic transmission, and neuron projection development (Figure 1C). These pathways are essential for proper neuronal communication, differentiation, and circuit formation during brain development. The downregulation of these genes following H3K27M loss suggests a potential shift away from an active neuronal or synaptic state. Significant alterations were also observed in antigen processing and presentation machinery (APPM)-related genes (Figure 1D), although the patterns of these changes were inconsistent across the two models, reflecting context-dependent regulatory effects of the mutation. Specifically, in SU-DIPG13 the KO line showed coordinated upregulation of HLA-A, HLA-B, HLA-C, B2M, TAP1, TAPBP, PSMB8, PSMB9 and the MHC class I transactivator NLRC5, whereas in BT245 the same genes were either modestly induced or unchanged, with HLA-A and HLA-B marginally reduced. These mRNA-level differences are consistent in direction with the surface HLA-I measurements reported below, but as shown subsequently they are not sufficient to predict the composition of the presented peptide repertoire, which diverged between parental and KO cells in both models.

### 3.2. Loss of H3K27M Alters HLA Class I Surface Expression in a Model-Dependent Manner

To determine whether the H3K27M mutation influences the HLA class I (HLA-I) APPM and alters the display of pHLA on DMG cell surfaces, we employed flow cytometry on paired model cell lines and assessed HLA-I expression using the pan-HLA-I antibody W6/32. Initially, we assessed the basal HLA-I expression levels in the parental BT245 and SU-DIPG13 cell lines (Figure 2A and Figure 2B, respectively). Flow cytometry analysis revealed that both cell lines were positive for cell surface HLA-I expression with no significant difference (*p* > 0.05) between two cell lines (Figure 2C). However, when we compared the parental cell lines with their paired H3K27M-KO counterparts, we observed that H3K27M-KO increased HLA-I expression in SU-DIPG13 cells by ~7-fold, whereas no change in HLA-I expression was observed in BT245 cells following H3K27M-KO (Figure 2C). We next investigated whether inflammatory cytokines enhance HLA-I expression in these cell lines and assessed whether the H3K27M mutation modulates the sensitivity of APPM to IFN-γ stimulation. Both parental BT245 and SU-DIPG13 cell lines showed a ~3- and 4-fold increase in HLA-I expression, respectively, following 48 h of treatment with 5 ng/mL IFN-γ (Figure 2D,E). Similarly, the H3K27M-KO cell lines showed HLA-I upregulation (~3- and ~2-fold, respectively) (Figure 2D,E). The divergent behaviour of the two models is notable: SU-DIPG13 showed a ~7-fold increase in surface HLA-I after loss of the mutant allele, whereas BT245 was unchanged. H3K27M is therefore not a uniform repressor of HLA-I in DMG, and its effect on antigen presentation is conditioned by tumour-intrinsic context. Several non-mutually exclusive factors may account for this divergence, including the differing anatomical origin and developmental lineage of the two lines (pontine versus thalamic), differences in baseline HLA-I and NLRC5 expression, co-occurring genetic alterations that differ between the models, and the extent to which PRC2 activity and H3K27me3 deposition are restored following loss of the mutant allele. These possibilities are considered further in the Discussion. Inspection of the RNA-seq data provides a mechanistic explanation for this divergence. In SU-DIPG13, loss of the mutant allele de-repressed the MHC class I axis in a coordinated manner, with NLRC5, the master transactivator of MHC class I, increased approximately 6-fold in the KO line, accompanied by increases in B2M (~5-fold), HLA-A (~4-fold), HLA-B (~22-fold), HLA-C (~9-fold), TAP1 (~3-fold), and the immunoproteasome subunits PSMB8 (~5-fold) and PSMB9 (~9-fold). In BT245, by contrast, NLRC5 induction was approximately 2.5-fold weaker, and the classical HLA genes responded discordantly, with HLA-C modestly increased in the KO line but HLA-A and HLA-B slightly reduced, so that no net change in surface HLA-I would be expected. The magnitude of NLRC5 de-repression following loss of the mutant allele therefore tracks closely with the magnitude of the surface HLA-I response measured by flow cytometry, indicating that the effect of H3K27M on antigen presentation is exerted largely through NLRC5-dependent transactivation and is contingent on how strongly that axis is released in a given cellular context.

### 3.3. The Loss of H3K27M Modulates the DMG Tumour Antigenic Landscape

Although we did not observe a consistent relationship between the H3K27M mutation and HLA-I expression, our transcriptomic analysis revealed higher expression of TAA-encoding genes in H3K27M-mutant cell lines compared to their KO counterparts. Specifically, 24% and 17% of the genes in our curated TAA list were significantly upregulated in H3K27M-mutant cells relative to their KO pairs (Figure 1A). Based on these findings, we sought to determine whether the H3K27M mutation influences the immunopeptidome repertoire of DMG cell lines by modulating TAA expression, thereby altering the antigenic landscape of these tumour cells. For clarity, throughout this manuscript “TAA gene” denotes a transcript from the curated list of 129 tumour-associated antigen source genes described in Section 2.9 and listed in Supplementary Table S1, which was assembled by a structured literature review of genes implicated in gliomagenesis and oncogenic signalling and cross-referenced against TCGA, the Human Protein Atlas cancer proteome and the Childhood Cancer Model Atlas. “TAA-derived peptide” or “TAA-derived pHLA” denotes an HLA-bound peptide identified by mass spectrometry whose source protein is encoded by one of these genes, and “TAA” alone refers to the source protein itself. TAA derivation was a predefined criterion for downstream candidate prioritisation rather than an independent discovery outcome.

To evaluate the presentation of pHLA derived from H3K27M-associated TAAs, we performed comprehensive immunopeptidomic analyses on parental and KO cell lines. Since IFN-γ treatment increases HLA-I expression across all cell lines, we also conducted immunopeptidomics analyses following IFN-γ stimulation. This approach allowed us to (a) increase the number of peptides identified from each cell line and (b) reduce the risk of classifying peptides as unique to a specific condition when non-detection under baseline conditions may reflect limited sampling depth or sensitivity of the mass spectrometry approach. Without IFN-γ treatment, such peptides might be missed, leading to an incomplete representation of the immunopeptidome. The overlap in pHLA repertoires between untreated and IFN-γ-treated conditions demonstrates that IFN-γ treatment expanded the pHLA repertoire. This expansion covers most peptides found in untreated conditions, especially in BT245, where the percentage of unique untreated peptides was minimal after stimulation (1.63% in parental and 1.59% in KO). In SU-DIPG13, IFN-γ had a less pronounced effect but increased the total number of peptides while maintaining a high proportion of shared peptides (63.03% in parental, 59.31% in KO) (Supplementary Figure S1). However, because IFN-γ is a biologically active perturbation that alters HLA-I expression and antigen processing, the untreated-versus-IFN-γ non-overlap cannot be interpreted as a direct estimate of DDA stochasticity, as it reflects both biological remodelling and analytical sampling. By combining peptides identified under both baseline and IFN-γ-treated conditions, we aimed to achieve a more comprehensive evaluation of the impact of the H3K27M mutation on the antigenic landscape of tumour cells.

In total, we identified 10,550, 10,107, 14,794, and 15,919 peptides from the BT245 parental, BT245 KO, SU-DIPG13 parental, and SU-DIPG13 KO cell lines, respectively (Supplementary Tables S3–S6). Of these, 41.15% of peptides in the BT245 pair and 51.84% in the SU-DIPG13 pair were shared between the parental and KO cell lines. Consistent with flow cytometry data, the BT245 parental cell line displayed a comparable or greater number of identified peptides than its KO counterpart, whereas the SU-DIPG13 KO cell line exhibited a higher number of peptides relative to its parental line (Figure 3A). Notably, the ~7-fold higher surface HLA-I of the SU-DIPG13 KO line translated into only ~1100 additional peptide identifications. This is expected, because the number of peptides identified by data-dependent immunopeptidomics is limited principally by the sequencing speed and dynamic range of the mass spectrometer rather than by the quantity of HLA available, and identifications approach saturation once the most abundant ligands are repeatedly selected for fragmentation. Increased HLA-I abundance is therefore reflected mainly in the signal intensity of individual ligands rather than in a proportional expansion of the identified repertoire, and peptide counts should be interpreted as a qualitative rather than a stoichiometric readout of HLA-I surface expression.

The majority of identified peptides fell within the canonical 8–12 amino acid range characteristic of HLA-I ligands, accounting for 87%, 77.7%, 86.8% and 91.1% of all peptides in BT245 parental, BT245 H3K27M-KO, SU-DIPG13 parental and SU-DIPG13 H3K27M-KO cells, respectively. Within the 8–12-aa peptide population, 9-mers represented the predominant peptide length across all four samples, accounting for 56.8%, 58.3%, 62.9% and 62.4% of peptides in BT245 parental, BT245 K27M-KO, SU-DIPG13 parental and SU-DIPG13 K27M-KO cells, respectively (Figure 3B). Binding predictions using NetMHCpan 4.1, based on the HLA genotyping results (Supplementary Table S7), revealed that over 80% of peptides in each sample were predicted to bind at least one HLA-I allele expressed by the respective cell line (Figure 3C). These findings validate the robustness and accuracy of the immunopeptidomics data, demonstrating that the identified peptides align within expected HLA-I binding characteristics. Furthermore, we identified 5584 unique protein sources in BT245 and 6933 in SU-DIPG13, providing a broad landscape of antigen presentation across these models.

In the BT245 parental cell line, we identified 97 TAA-derived pHLA from 47 TAA source proteins, accounting for 1.1% of the total immunopeptidome. In contrast, the KO counterpart exhibited 58 pHLA from 28 TAA source proteins, representing 0.8% of the immunopeptidome. Similarly, in SU-DIPG13, the parental line contained 133 pHLA from 53 TAA source proteins, comprising 1.1% of the total immunopeptidome, whereas the KO variant displayed 137 pHLA from 45 TAA source proteins, corresponding to 1.0% of the immunopeptidome. We further investigated whether specific TAAs contributed peptides detected only in the parental cell lines and not in the matched K27M-KO counterparts. In the BT245 pair, 22 TAA source proteins yielded 34 peptides detected only in the parental line (Figure 4A), whereas in SU-DIPG13, 14 TAA source proteins yielded 20 peptides detected only in the parental cells (Figure 4B). Notably, seven peptides derived from shared TAAs were identified in both BT245 and SU-DIPG13 and were restricted to HLA-A*01:01. These findings suggest that the H3K27M mutation regulates the presentation of specific TAAs, leading to differential peptide presentation and shaping the immunopeptidome repertoire in an HLA allele-dependent manner.

To identify candidate peptides for further validation and functional assays, we applied elaborate selection criteria to the 54 candidate peptides, prioritising a subset of 10 peptides based on their immunogenic potential. These ten selected peptides spanned five HLA types and comprised four BT245-specific peptides, two SU-DIPG13-specific peptides and four peptides detected in both models (Table 1). Peptide 5 (KPNHPLLQA) carries a C-terminal alanine, which is atypical for most B7-supertype allotypes but is compatible with the reported HLA-B*56:01 motif, together with proline at P2; the peptide was predicted to bind strongly (%Rank_EL 0.032), although peptide–HLA stability was not measured experimentally. Structural modelling of the ten prioritised peptide–HLA complexes are shown in Supplementary Figure S3. The expression of the corresponding source genes was also examined relative to their H3K27M-KO counterparts in the isogenic RNA-seq data (Supplementary Table S8). With the exception of CBX2 in BT245, all prioritised source genes showed higher mean expression in the parental cells, although the magnitude of these differences varied widely, with the strongest consistent parental enrichment observed for KIT and XKR5. This variability indicates that differential peptide presentation does not show a uniform quantitative relationship with source-gene transcription. To further assess tumour-associated expression, we examined the selected source genes in the CCMA paediatric DMG and non-malignant cohorts (Supplementary Table S9). Seven of the eight source genes showed higher median expression in H3K27-DMG than in the non-malignant cohort; KIT was the exception, being enriched in the parental cell lines but not in patient tumours, which further indicates that source-gene transcription in cell line models is an imperfect predictor of tumour-associated presentation in vivo. RNA expression of the selected source genes was also assessed in available DMG H3K27M autopsy samples to support their relevance to primary disease (Supplementary Figure S4).

First, to validate the peptide sequence assignments, we synthesised the selected peptides and performed MS2 spectral comparisons between the eluted peptides and their synthetic counterparts, confirming sequence fidelity (Supplementary Figure S2). Additionally, comparison with publicly available immunopeptidomics datasets confirmed that these peptide sequences were not detected among previously reported HLA-bound peptides derived from benign human tissues [29]. Because absence from a reference atlas is only informative for allotypes that the atlas actually sampled, we examined which of the restricting allotypes are represented in the normal brain and cerebellum subsets of the HLA Ligand Atlas. HLA-A*01:01 (peptides 1, 2 and 7–10), HLA-B*08:01 (peptide 3) and HLA-A*68:02 (peptide 4) are represented in those subsets, so non-detection of the corresponding peptides carries some negative weight. HLA-A*31:01 (peptide 6) and HLA-B*56:01 (peptide 5) are not represented, and for these two peptides non-detection in the Atlas is uninformative. HLA-A*31:01 belongs to the A3 supertype and shares a preference for basic C-terminal residues with A3-supertype allotypes that are represented in the brain subset, which provides only indirect reassurance; for HLA-B*56:01 no closely motif-related allotype is represented, and we therefore draw no inference from its absence. We next assessed normal-brain expression using GTEx RNA datasets, which showed almost no expression of XKR5, CBX2, KIT, and PRAME, and variable expression of SLITRK2, PTPRU, ATP1A3, and ST3GAL6 in the midbrain region (Supplementary Table S10). At the protein level, HPA provided no antibody-based expression data for the selected source genes in the normal midbrain, so no protein-level conclusion can be drawn for these regions.

### 3.4. H3K27M-Associated Peptides Elicit Peptide-Specific Cytotoxic T Cell Responses

As a therapeutically relevant readout, peptide immunogenicity was functionally assessed by investigating their ability to elicit strong and specific cytotoxic T cell responses in vitro. To this end, we used viably frozen PBMCs from 9 healthy donors expressing HLA types (HLA-A*01:01, A*31:01, B*08:01, A*68:02, and B*56:01) matching those of the peptides identified in DMG cells. PBMCs from 3 HLA-matched healthy donors/peptides were cultured in the presence of each of the 10 selected DMG peptides for 2 weeks. T cells were then investigated for their ability to specifically lyse autologous PHA T blasts unpulsed or pulsed with each of the relevant peptides using standard cytotoxicity assays. As shown in Figure 5A and 6 peptides (peptides 1–6) were immunogenic, as T cell cultures demonstrated the ability to specifically kill peptide-pulsed PHA T blasts with a significantly higher specific lysis at 20:1 and 10:1 effector:target ratios as compared to unpulsed targets in all cases. Limited donor-to-donor variation was observed among the three donors tested, as reflected by the SEM values.

In contrast, 4 peptides (peptides 7–10) did not elicit detectable peptide-specific cytotoxicity against peptide pulsed autologous PHA blasts, again with similar results obtained with PBMCs from the three donors tested. The ability of PBMCs of the same donors to generate T cells specific for an unrelated HLA-matched immunogenic peptide (HLA-A*01:01 Flu NP epitope) ensured the functionality of these cells and served as a positive control for T-cell responsiveness.

T cells specific for the 6 immunogenic peptides were also investigated for their ability to specifically lyse parental and H3K27M-KO DMG cell lines. As shown in Figure 5B, with the exception of T cells specific for peptide 5, all T cell cultures exerted significantly higher specific cytotoxicity against the parental as compared to the corresponding H3K27M-KO DMG cell lines. This was particularly notable in SU-DIPG13, where, despite the higher surface HLA-I expression of the H3K27M-KO cells described above (Figure 2C), T cells specific for peptides 3 and 4 preferentially killed the parental cells. Notably, in all cases, the extent of killing of the H3K27M-KO cell lines was similar to that observed against the K562 cells included as a control of non-specific lysis. These findings indicate that T cells specific for five of the six immunogenic peptides preferentially recognise and kill the parental cells compared with the corresponding H3K27M-KO cell lines, consistent with the lack of detection of these peptides in the immunopeptidome of these cells. Importantly, the DMG target cells used in these cytotoxicity assays were cultured under basal conditions and were not treated with IFN-γ, whereas the peptides themselves were originally identified in IFN-γ-stimulated parental cells (condition of detection for each peptide is given in Table 1). The ability of peptide-specific T cells to lyse untreated parental DMG cells therefore demonstrates that these peptides are presented constitutively at densities sufficient for T cell recognition, even where their abundance in unstimulated cells fell below the detection limit of data-dependent mass spectrometry. This provides functional evidence that the candidates identified under IFN-γ stimulation are not artefacts of cytokine-induced antigen processing, and that IFN-γ stimulation served here to increase the depth of peptide discovery rather than to create a presentation state absent from untreated cells.

These results demonstrate that the identified immunogenic peptides elicit T-cell responses capable of specifically recognising and lysing H3K27M-mutant DMG cells. The reduced recognition of the KO cell lines indicates that presentation of five of these tumour-associated peptides is associated with the presence of the mutant H3F3A allele. As the peptides derive from non-mutated source proteins, they represent epigenetically dysregulated tumour-associated antigens rather than neoantigens and are candidate targets for TAA-directed T cell therapy.

## 4. Discussion

DMG remains one of the most aggressive paediatric brain tumours, with no effective standard of care beyond palliative radiotherapy [1,23,36,37]. The failure of chemotherapy and targeted therapies underscores the urgent need for precision medicine approaches guided by a deeper understanding of tumour biology. While DMG was initially classified within the glioblastoma (GBM) spectrum, mounting evidence has established pDMG-H3K27-altered as a distinct entity, differing from adult GBMs in its clinical presentation, developmental origins, and tumour microenvironment [38,39]. The discovery of H3K27M as a recurrent driver mutation has been pivotal in understanding the biology of DMG, as it fundamentally reprograms the epigenetic landscape. H3K27M disrupts PRC2-mediated H3K27me3, resulting in widespread transcriptional dysregulation and stalled differentiation pathways, which collectively sustain tumour growth and impact antigen presentation [4,6,7,11,40,41].

Given that these epigenetic changes extend to antigen processing, we hypothesised that H3K27M could reshape the tumour’s antigenic landscape, influencing immune recognition and susceptibility to immunotherapy. To address this, we leveraged pDMG-H3K27M-KO models to investigate how H3K27M-driven epigenetic dysregulation modulates HLA-I antigen presentation and tumour cell antigenicity, and to identify actionable immunotherapeutic targets.

Our findings indicate that H3K27M alters antigen processing and presentation in a context-dependent manner. While PRC2 repression via EZH2, EED, and SUZ12 inhibition has been shown to de-repress MHC-I antigen presentation genes [41,42], our analyses in isogenic pDMG models (parental H3K27M-mutant and H3K27M-KO variants of SU-DIPG13 and BT245) reveal a more complex regulatory network. Flow cytometry demonstrated that H3K27M knockout led to increased HLA-I expression in SU-DIPG13 cells, whereas no significant change was observed in BT245 cells, suggesting that antigen presentation is regulated by additional factors beyond PRC2 inhibition. Interestingly, both parental and KO cells retained intact IFN-γ inducibility, supporting the feasibility of cytokine-based immunotherapies to enhance antigen presentation in DMG. Immunopeptidomic profiling further highlighted variability between models, with BT245 parental cells presenting a higher number of peptides than their KO counterparts, while SU-DIPG13 showed the contrary. This distinction was also reflected functionally, as despite higher surface HLA-I expression in SU-DIPG13 H3K27M-KO cells, peptide 3- and 4-specific T cells preferentially recognised the parental cells, indicating that greater overall HLA-I expression does not necessarily translate into greater antigen-specific tumour-cell immunogenicity. This supports the notion that H3K27M’s influence on antigen presentation is modulated by tumour-intrinsic factors, such as lineage specification, differentiation status, and chromatin regulatory mechanisms. These findings are consistent with previous reports showing that the effects of H3K27M and H3K27me3 distribution are highly dependent on cellular context and neurodevelopmental timing, further highlighting the complexity of its role in antigen presentation [7,41,43].

Our data revealed significantly higher expression of multiple TAA genes in the parental H3K27M-mutant models (Figure 1A), indicating that their expression is associated with the presence of the mutant allele. Consistently, our immunopeptidomics analysis identified numerous pHLA complexes, including a substantial fraction of novel peptides absent from the Immune Epitope Database (IEDB) [44], expanding the catalogue of DMG-associated antigens. In the combined pHLA repertoire from the BT245 parental and KO datasets, 7944 peptides were previously documented in IEDB and 2439 were not documented in IEDB, of which 1372 were detected exclusively in the parental cells. In the SU-DIPG13 model, we identified 4146 novel sequences and 864 unique to parental cells, with differences in the composition of the TAA-derived peptide repertoire between parental and KO cells in both models (Figure 4). The TAA-derived fraction of the total immunopeptidome was largely unchanged in SU-DIPG13 (1.1% parental versus 1.0% KO) and lower in the BT245 KO line (1.1% versus 0.8%), so these differences reflect antigen-selective, model-dependent changes in which TAAs are presented rather than a global shift in TAA-derived peptide abundance.

We selected 10 candidate peptides for subsequent immunogenicity assays to distinguish therapeutically relevant targets from those that are merely presented. Although we identified four shared peptides across both models, derived from the source antigens: cKIT, PTPRU, ATP1A3, and ST3GAL6, these peptides failed to induce T cell responses. This lack of T cell activation may result from low intrinsic immunogenicity or insufficient TCR affinity, underscoring the necessity for functional validation when selecting TAAs for immunotherapy. Beyond the broad effects on antigen presentation, the study identified six immunogenic peptides from four previously uncharacterized TAAs in DMG: SLITRK2, PRAME, XKR5, and CBX2 (Table 1). Among the identified peptides, only one peptide (Peptide 4, derived from CBX2), had been previously documented in the IEDB as a naturally presented ligand associated with cervical cancer and acute lymphoblastic leukemia under HLA-I restriction [45,46]. These peptides were validated using functional T cell assays, demonstrating their ability to expand T cells from HLA-matched donor PBMCs and elicit peptide-specific cytotoxic responses. Of note, CBX2, a key component of the PRC1 complex, plays a crucial role in maintaining transcriptional repression through chromatin remodelling and histone modification. By binding to H3K27me3, it modulates gene expression and lineage differentiation [47,48,49]. The identification of immunogenic CBX2-derived peptide in the DMG immunopeptidome underscores the direct role of epigenetic dysregulation and chromatin regulators in shaping the tumour antigenic landscape, highlighting their potential as novel targets for immunotherapy.

Additionally, PRAME, a well-characterised cancer-testis antigen, is particularly promising, given its restricted expression in normal tissues and high prevalence in multiple cancers [50,51,52]. Current efforts focus on PRAME-specific immunotherapies, such as TCR-engineered T cell therapy (TCR-T), which has shown promising clinical responses in other malignancies [53]. Furthermore, previous studies have reported presentation of PRAME-derived pHLA complexes in distinct patient-derived DMG cell lines, highlighting its relevance as a target antigen in H3K27M-DMG [21].

Despite the insights gained from this study, several limitations should be acknowledged. Importantly, normal-tissue safety remains a key consideration. Although the prioritised peptides were not detected in the HLA Ligand Atlas, this resource is derived from adult donors and provides limited brain sampling, making it an imperfect reference for paediatric DMG. This is particularly relevant for SLITRK2, which shows appreciable normal-brain RNA expression, while the neuronal expression of ATP1A3 similarly warrants caution. Direct validation of these pHLA complexes in relevant normal paediatric brain tissue and appropriate neurotoxicity assessment would therefore be required before therapeutic translation. This caution applies most sharply to SLITRK2, which contributes three of the six immunogenic peptides identified here. SLITRK2 is a neuronal transmembrane protein of the SLIT/NTRK-like family with largely brain-restricted expression, and both GTEx and HPA record appreciable SLITRK2 transcript in normal brain (Supplementary Table S10), while no antibody-based protein data are available for the relevant regions. T cells directed at SLITRK2-derived epitopes therefore carry a plausible on-target, off-tumour risk in normal central nervous system tissue, and this risk cannot be excluded on the basis of the immunopeptidomic reference data used here: the HLA Ligand Atlas samples brain among its lowest-yielding tissues, draws on adult autopsy donors, and includes neither paediatric pons or thalamus nor the peritumoural brain that infused T cells would encounter. The same consideration applies to ATP1A3, a neuron-specific Na+/K+-ATPase subunit whose loss of function causes alternating hemiplegia of childhood and rapid-onset dystonia–parkinsonism, and which contributed two of the peptides that proved non-immunogenic here. Direct interrogation of paediatric normal brain immunopeptidomes, together with preclinical neurotoxicity testing, would be a prerequisite for clinical development of the SLITRK2 epitopes in particular. More broadly, our findings are based on in vitro models, and while isogenic H3K27M-KO cell lines provide a controlled system to dissect the effects of H3K27M on antigen presentation, they do not fully recapitulate the complexity of the in vivo tumour microenvironment. The immune landscape of DMG is influenced by factors such as stromal interactions, local immunosuppression, and blood–brain barrier constraints [9,12,13,14,15,54], which may further modulate antigen processing and presentation in a physiological setting. Additionally, our immunopeptidomic analysis was conducted on two DMG cell lines, which, while representative, do not capture the full heterogeneity observed across patient-derived tumours. While we identified novel immunogenic peptides, their functional impact on tumour immune responses and therapeutic efficacy in patients remains to be explored. Future studies should prioritise comprehensive immunogenicity assessments, including tumour-infiltrating lymphocyte responses and in vivo tumour clearance assays, to determine their clinical applicability. A further consideration is that immunopeptidomic profiling was performed under both basal and IFN-γ-stimulated conditions. IFN-γ substantially remodels antigen processing, inducing the immunoproteasome subunits PSMB8, PSMB9 and PSMB10 as well as TAP1 and TAP2, so peptides detected only after stimulation may not be constitutively presented by DMG cells in vivo. While the cytokine-rich milieu of an inflamed or immunotherapy-treated tumour may recapitulate aspects of this state, constitutive presentation of prioritised candidates in situ requires confirmation. In addition, our database search was restricted to canonical open reading frames. Because H3K27M-driven epigenetic dysregulation is known to de-repress cryptic transcription, including endogenous retroviral elements, a proportion of the DMG immunopeptidome is likely to derive from non-canonical ORFs; these were not interrogated here, and proteogenomic re-analysis of the deposited spectra against a non-canonical ORF database represents a clear next step that would further expand the antigenic landscape described. A further dimension not addressed here is HLA class II presentation and the CD4+ T cell compartment. Our workflow used the pan-HLA class I antibody W6/32, so every peptide reported in this study is an HLA class I ligand and the data are silent on the class II immunopeptidome of these models. That omission carries particular weight in DMG. Efforts to target the H3K27M neoepitope through HLA class I have been inconclusive, including for the HLA-A*02:01-restricted peptide [19,20], whereas the mutation-specific peptide vaccine evaluated in adults with H3K27M DMG acts through MHC class II: vaccine-induced responses were mediated by CD4+ T cells, were suppressed by MHC class II- but not MHC class I-blocking antibodies, and were observed across multiple HLA types [23], consistent with the original identification of K27M-mutant histone H3 as a class II-restricted target [55]. CD4+ T cells also contribute to antitumour immunity indirectly, through licensing of dendritic cells, provision of help to CD8+ T cells, and cytokine-mediated remodelling of the tumour microenvironment, which is relevant in an immunologically cold tumour such as DMG. Whether H3K27M remodels the class II immunopeptidome as it does the class I repertoire described here is therefore an open and clinically consequential question, and one our data cannot address. Two features of the disease shape how it should be approached: DMG cells express little constitutive HLA class II, so class II ligands in situ would derive substantially from professional antigen-presenting cells that have acquired tumour material, and interrogation of tumour-intrinsic class II presentation in vitro generally requires cytokine induction or enforced expression of the class II transactivator CIITA. The same isogenic models and the same semi-automated workflow used here could be applied with a pan-HLA class II antibody to address this directly, and we regard it as the most informative single extension of the present study. Finally, our immunopeptidomic data is qualitative, being acquired by data-dependent acquisition and compared on the basis of peptide identification rather than abundance. As an additional re-interrogation of the existing raw data, MS1 extracted-ion chromatograms showed precursor-level evidence for FSDLKSPLHY in the H3K27M-KO samples of both BT245 and SU-DIPG13, and for LTDPEVTNY in SU-DIPG13 KO, for which corresponding MS2 identifications were not observed. For the remaining prioritised peptides, no corresponding MS1 precursor evidence was observed in the matched H3K27M-KO samples. Quantitative strategies, such as DDA library generation combined with data-independent acquisition or isobaric labelling, would permit differential presentation to be assessed statistically and expressed as fold-change, and would strengthen conclusions drawn from parental-versus-knockout comparisons. Reassuringly on this point, the six immunogenic peptides were identified in IFN-γ-stimulated cells, yet their cognate T cells lysed untreated parental DMG cells, indicating that these candidates are constitutively presented and that IFN-γ acted to extend detection depth rather than to generate a non-physiological repertoire. The same cannot be assumed for peptides detected only after stimulation and not functionally tested, and these should be regarded as provisional pending confirmation under basal conditions.

Taken together, our study establishes a link between H3K27M-driven transcriptional dysregulation and tumour antigenicity, highlighting the potential of immunopeptidomics as a tool for identifying candidate HLA-restricted targets for further therapeutic investigation. By leveraging epigenetically dysregulated antigens, our findings identify distinct antigenic vulnerabilities that support further evaluation in T cell-based immunotherapy. Future studies should further evaluate the therapeutic potential of H3K27M-associated antigens, particularly in the context of adoptive T cell therapies and multi-epitope cancer vaccines. These findings lay the foundation for precision immunotherapy approaches in DMG, offering new hope for treating this otherwise intractable disease.

## 5. Conclusions

In conclusion, our study provides new insights into the immunopeptidome of H3K27M-DMG, revealing how epigenetic dysregulation influences antigen presentation and immune recognition. By leveraging immunopeptidomics and functional immunology, we identified TAA-derived epitopes detected in H3K27M-mutant cells but not in their matched mutant-allele-knockout counterparts, and functionally validated their immunogenicity. Our findings highlight PRAME and other dysregulated antigens as promising candidate targets for HLA-restricted T-cell therapies, underscoring the feasibility of precision immunotherapy approaches in DMG. While further validation in patient-derived and in vivo models is necessary, our study establishes a foundation for antigen-directed immunotherapy strategies in this otherwise treatment-refractory disease. Future efforts should focus on integrating multi-epitope targeting, immune checkpoint modulation, and cytokine-based adjuvants to enhance therapeutic efficacy. These findings pave the way for the next generation of personalised immunotherapies aimed at improving outcomes for patients with H3K27M-DMG.

## Figures and Tables

**Figure 1:**
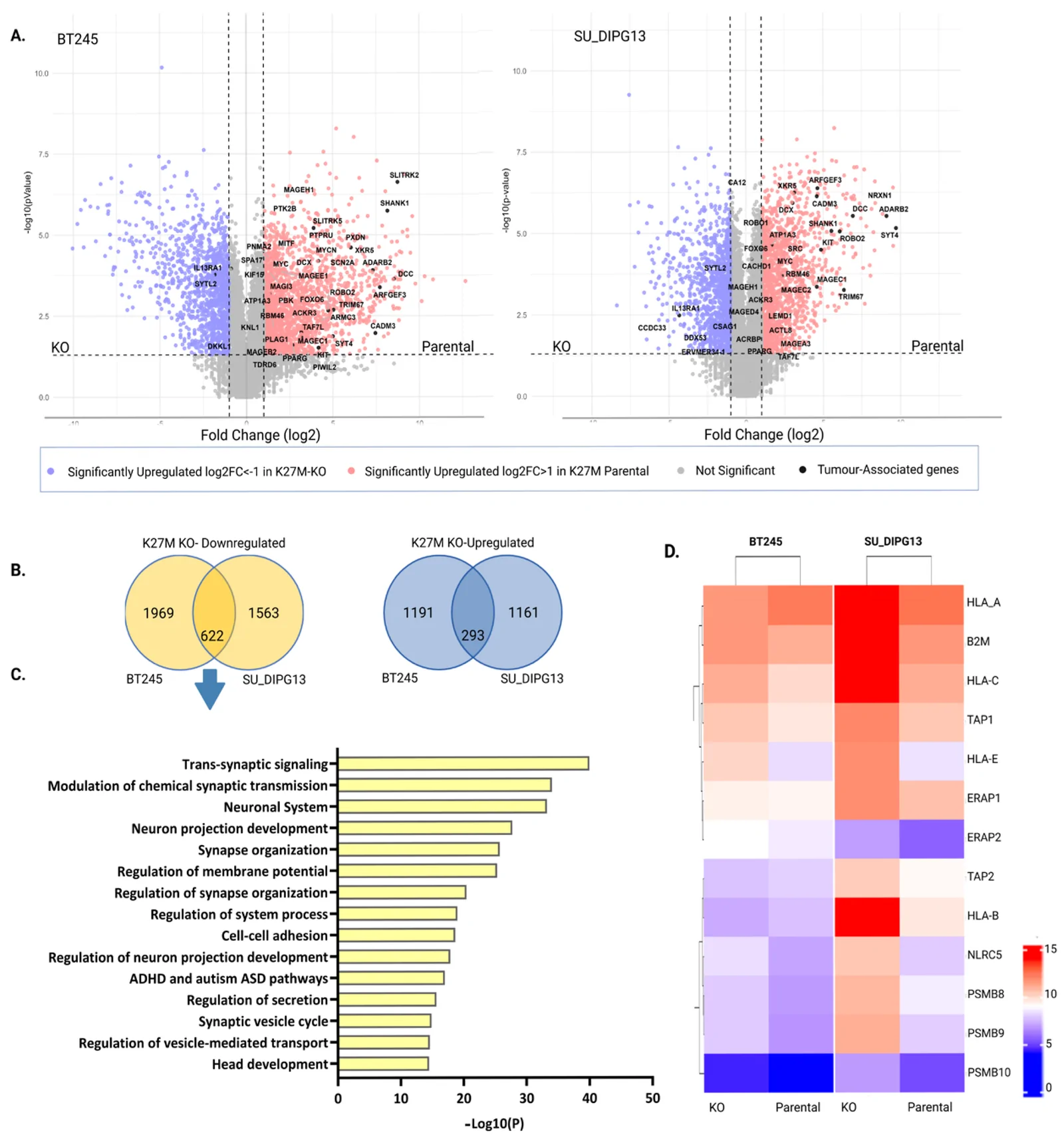
Transcriptomic analysis of isogenic parental H3K27M-mutant and H3K27M-KO models of Diffuse Midline Glioma (DMG). Parental DMG lines carry one H3.3K27M-mutant and one wild-type H3F3A allele; CRISPR-Cas9 editing disrupted the mutant allele only, leaving the wild-type allele and all other histone H3 genes intact, so the H3K27M-KO lines retain wild-type histone H3.3 expression without the mutant H3.3K27M allele. These are mutant-allele knockouts, not histone H3 knockouts. (**A**) Differential gene expression (log2 fold change) and statistical significance (−log10 *p*-value) in BT245 (**left**) and SU-DIPG13 (**right**), with TAA-encoding genes highlighted. (**B**) Number of shared and unique differentially expressed genes between BT245 and SU-DIPG13 parental and KO pairs (|log2FC| > 1, adjusted *p* value < 0.05). (**C**) The top 15 enriched gene ontology terms for shared downregulated genes after H3K27M-KO. (**D**) Differential expression of genes associated with APPM, between parental H3K27M-mutant and H3K27M-KO cells for both cell lines. In all panels, KO denotes CRISPR-mediated knockout of the mutant H3F3A allele only; the wild-type H3F3A allele is retained, and the KO lines continue to express wild-type histone H3.3.

**Figure 2:**
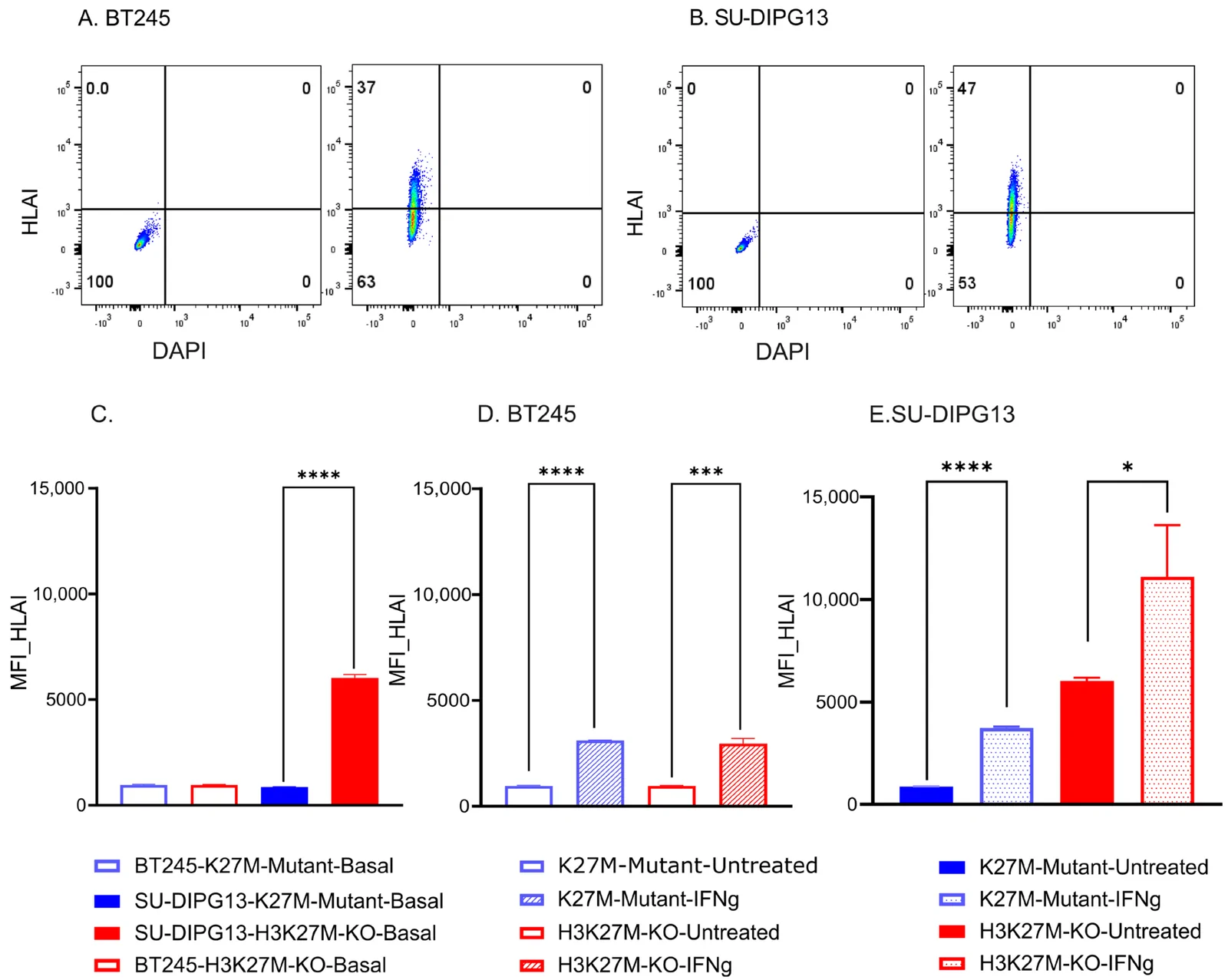
HLA-I expression in parental H3K27M-mutant and H3K27M-KO DMG cell lines. (**A**,**B**) Representative flow cytometry dot plots for parental H3K27M-mutant BT245 and SU-DIPG13 cell lines under unstained and basal conditions. The *X*-axis represents DAPI, indicating the live cell population, while the *Y*-axis represents HLA-I expression. The upper left quadrant indicates HLA-I-positive cells. Percentages reflect the proportion of cells within each quadrant. Colours in the pseudocolour plots represent event density, with warmer colours indicating higher event density. (**C**) Comparison of mean fluorescence intensity (MFI) of HLA-I expression in basal parental H3K27M-mutant and H3K27M-KO BT245 and SU-DIPG13 cell lines. (**D**) BT245 parental H3K27M-mutant and H3K27M-KO cells each showed approximately three-fold upregulation of HLA-I expression following 48-h treatment with 5 ng/mL IFN-γ. (**E**) SU-DIPG13 parental H3K27M-mutant and H3K27M-KO cells showed approximately four-fold and two-fold IFN-γ-induced upregulation of HLA-I expression, respectively. (* *p* < 0.05, ** *p* < 0.01, *** *p* < 0.001, **** *p* < 0.0001).

**Figure 3:**
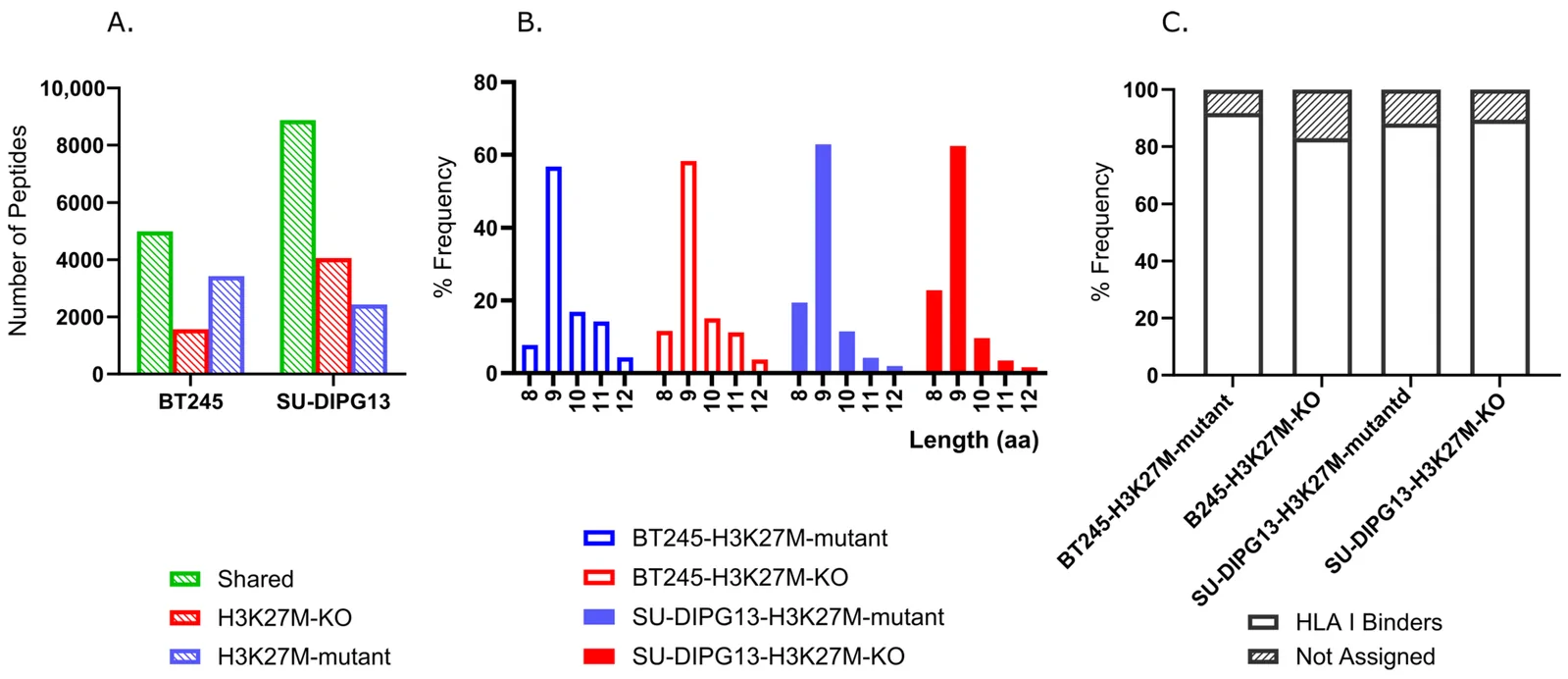
Comparative analysis of HLA-I peptides in BT245 and SU-DIPG13 parental H3K27M-mutant and H3K27M-KO cell lines. (**A**) Number of HLA-I peptides identified in BT245 and SU-DIPG13 H3K27M-mutant and H3K27M-KO cell lines, classified as detected only in H3K27M-mutant cells, detected only in H3K27M-KO cells, or shared peptides. (**B**) Length distribution of HLA-I peptides in BT245 and SU-DIPG13. (**C**) Proportion of peptides assigned as binder to at least one HLA-I allele in BT245 and SU-DIPG13 cell lines.

**Figure 4:**
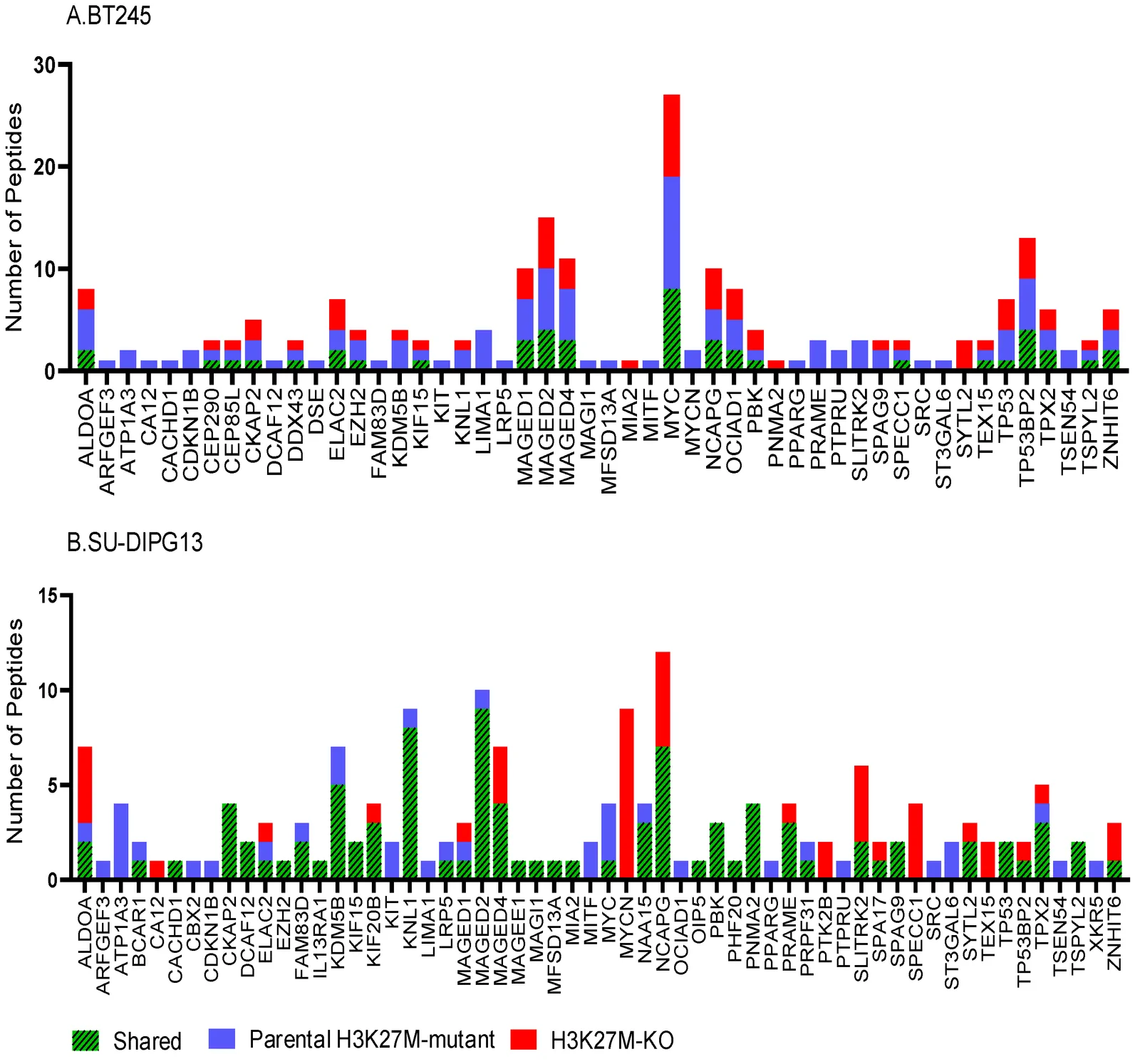
Contribution of Tumour-Associated Antigens (TAAs) to the antigenic landscape in (**A**) BT245 and (**B**) SU-DIPG13 H3K27M-mutant and H3K27M-KO models. Bar graph shows the total number of HLA-I peptides derived from selected TAAs. Peptides are categorised as detected only in H3K27M-mutant cells (blue), detected only in H3K27M-KO cells (red), and shared (green).

**Figure 5:**
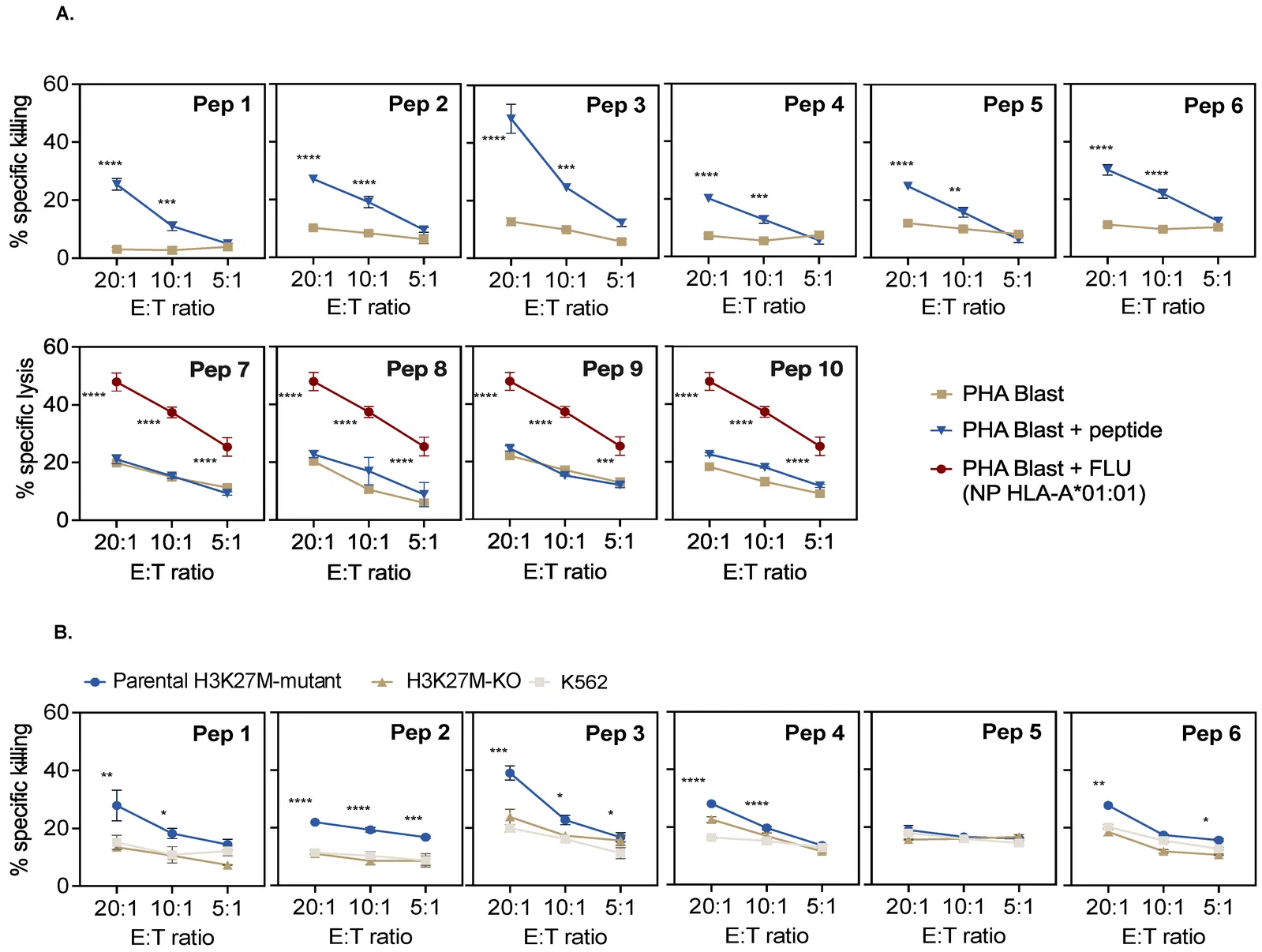
(**A**) Functional validation of the immunogenicity of peptides derived from H3K27M-DMG and identified by immunopeptidomics. Peripheral blood mononuclear cells from 3 unrelated HLA-matched healthy donors were cultured for 14 days in the presence of each peptide. Expanded T cells were then assessed for their ability to lyse autologous PHA T blasts loaded or not with the corresponding peptides at the indicated effector:target ratios. For the 4 non-immunogenic HLA-A01:01 peptides (Pep 7–Pep 10), the graphs also report the generation of T cells specific for the Flu NP HLA-A01:01 epitope to confirm T-cell functionality. (**B**) In vitro cytotoxicity of T cells specific for the 6 immunogenic peptides against BT245 (Pep1, 2, 5, and 6) or SU-DIPG13 cells (Pep3 and 4). The cytotoxic responses of the same T-cell cultures against K562 cells are also included as a control for non-specific killing. Data are presented as mean ± SEM from three independent HLA-matched healthy donors, with the error bars representing the SEM across donors. Statistics: Two-way ANOVA, Fisher’s least significant difference test for post hoc comparisons with negative controls; * *p* < 0.05; ** *p* < 0.01; *** *p* < 0.001; **** *p* < 0.0001.

**Table 1:** Peptides prioritised for validation and immunogenicity testing. Peptides were selected based on detection in parental cells and not in the matched H3K27M-KO cells, association with tumour-associated antigens (TAAs), and predicted binding to expressed HLA alleles. Details include source proteins, associated cell models, HLA types, peptide lengths, detection conditions, and NetMHCpan %Rank_EL binding predictions.

Peptide Number	Peptide Sequence	Source Protein	Peptide Length	Cell Model	HLA Allele	Condition of Detection	NetMHCpan %Rank_EL
1	ATPREPELLY	SLITRK2	10	BT245	A01:01	IFN-γ, BT245 parental H3K27M-mutant	0.125
2	NLDVSSFQLQY	SLITRK2	11	BT245	A01:01	IFN-γ, BT245 parental H3K27M-mutant	0.052
3	HWDAALTSL	XKR5	9	SU-DIPG13	B08:01	IFN-γ, SU-DIPG13 parental H3K27M-mutant	0.371
4	FVTDVTANL	CBX2	9	SU-DIPG13	A68:02	IFN-γ, SU-DIPG13 parental H3K27M-mutant	0.022
5	KPNHPLLQA	SLITRK2	9	BT245	B56:01	IFN-γ, BT245 parental H3K27M-mutant	0.032
6	RLWGSIQSR	PRAME	9	BT245	A31:01	IFN-γ, BT245 parental H3K27M-mutant	0.015
7	ASDPAVPCEY	PTPRU	10	Shared	A01:01	IFN-γ (BT245); basal (SU-DIPG13); both parental H3K27M-mutant	0.008
8	LTDPEVTNY	KIT	9	Shared	A01:01	IFN-γ (BT245); basal (SU-DIPG13); both parental H3K27M-mutant	0.001
9	ETEDPNDNRY	ATP1A3	10	Shared	A01:01	IFN-γ, both parental H3K27M-mutant lines	0.009
10	FSDLKSPLHY	ST3GAL6	10	Shared	A01:01	IFN-γ, both parental H3K27M-mutant lines	0.002

## Data Availability

The mass spectrometry proteomics data generated in this study have been deposited to the ProteomeXchange Consortium via the PRIDE partner repository under accession PXD061731 (DOI: 10.6019/PXD061731). Annotated MS2 spectra for all peptides selected for synthesis and functional validation, together with their synthetic peptide comparisons, are provided in Supplementary Figure S2. All additional datasets supporting the findings of this study are provided in the Supplementary Data Excel file. Further information is available from the corresponding authors upon reasonable request.

## Supplementary Materials

The following supporting information can be downloaded at https://www.mdpi.com/article/10.3390/cancers18193142/s1: Figure S1: Comparison of pHLA Repertoires in Isogenic H3K27M-DMG Models Before and After IFN-γ Treatment. Figure S2: MS2 spectral comparisons between eluted and synthetic peptides. Figure S3: (A–J): Peptides #1–10 prioritised for structural validation and immunogenicity testing. Peptides were selected based on detection in H3K27M parental cells and not in the matched knockout models, and annotated association with known tumour-associated antigens (TAAs). Left: Predicted 3D structure of the HLA–peptide complex rendered in PyMOL. Right: Structural confidence metrics and modelled peptide placement. Figure S4: RNA expression of candidate pHLA source genes in DIPG H3K27M autopsy samples. RNA expression values are log2(tpm +1) with variance-stabilising transformation applied from the DESeq2 R package. Expression values for genes in samples for which detected TPM are zero before transformation were not plotted, and a dashed line is shown at this lower limit of detection to aid interpretation. Table S1: Curated tumour-associated antigen (TAA) gene list. Table S2: RNA-seq gene expression matrix. Table S3: BT245 immunopeptidome. Table S4: BT245 NetMHCpan binder predictions. Table S5: SU-DIPG13 immunopeptidome. Table S6: SU-DIPG13 NetMHCpan binder predictions. Table S7: HLA class I genotypes of the cell line models. Table S8: Source-gene CPM values in parental and H3K27M-KO models. Table S9: Gene expression in CCMA H3K27-DMG and non-malignant cohorts. Table S10: HPA/GTEx normal-brain expression of selected source genes.
