# Supplementary material for: H3K27M Mutation Modulates the Antigenic Landscape in Diffuse Midline Glioma
Source: Cancers (Basel). 2026 Sep 28;18(19):3142. doi: 10.3390/cancers18193142 (PMC13664579; doi:10.3390/cancers18193142)
Supplement: Supplementary file 1 [file cancers-18-03142-s001.zip › SupplementaryFigures.pdf]

# H3K27M Mutation Modulates the Antigenic Landscape in Diffuse Midline Glioma

**Tima Shamekhi [1,2], Bijun Zeng [3,4], Claire Xin Sun [1], Paul Daniel [1], Terry C. C. Lim Kam Sian [1,2,5], Gabriel Goncalves [1,2,5], Grace Huang [1,2], Farnaz Fahimi [1,2], Nivedhitha Selvakumar [1,5], Erwin Tanuwidjaya [1,2,5], Isaac Woodhouse [1,5], Bettina Kritzer [6], Ralf B. Schittenhelm [5], Roberta Mazzieri [3,4], Jason E. Cain [1,7,8], Javad Nazarian [6], Jordan R. Hansford [9], Ron Firestein [1], Riccardo Dolcetti [3,4,10,*] and Pouya Faridi [1,2,5,*]**

1 Centre for Cancer Research, Hudson Institute of Medical Research, Clayton, VIC 3168, Australia; tima.shamekhi@monash.edu (T.S.); claire.sun@hudson.org.au (C.X.S.); paul.daniel@hudson.org.au (P.D.); terry.lim@monash.edu (T.C.C.L.K.S.); gabriel.goncalves@monash.edu (G.G.); grace.huang@monash.edu (G.H.); farnaz.fahimi@monash.edu (F.F.); nivedhitha.selvakumar@monash.edu (N.S.); erwin.tanuwidjaya1@monash.edu (E.T.); isaac.woodhouse@monash.edu (I.W.); jason.cain@hudson.org.au (J.E.C.); ron.firestein@hudson.org.au (R.F.)
2 Department of Medicine, School of Clinical Sciences, Monash University, Clayton, VIC 3168, Australia
3 Peter MacCallum Cancer Centre, Melbourne, VIC 3000, Australia; bijun.zeng@petermac.org (B.Z.); roberta.mazzieri@petermac.org (R.M.)
4 Sir Peter MacCallum Department of Oncology, The University of Melbourne, Parkville, VIC 3010, Australia
5 Monash Proteomics and Metabolomics Platform, Biomedicine Discovery Institute, Monash University, Clayton, VIC 3800, Australia; ralf.schittenhelm@monash.edu
6 Department of Paediatrics, University Children's Hospital Zurich, 8032 Zurich, Switzerland; bettina.kritzer@kispi.uzh.ch (B.K.); javad.nazarian@uzh.ch (J.N.)
7 Department of Molecular and Translational Science, School of Medicine, Nursing and Health Sciences, Monash University, Clayton, VIC 3800, Australia
8 Department of Paediatrics, Monash University, Clayton, VIC 3168, Australia
9 Michael Rice Centre for Hematology and Oncology, Women's and Children's Hospital, South Australia Health and Medical Research Institute, South Australia Immunogenomics Cancer Institute, Adelaide University, Ade-laide, SA 5000, Australia; jordan.hansford@sa.gov.au
10 Department of Microbiology and Immunology, The University of Melbourne, Parkville, VIC 3010, Australia
* Correspondence: riccardo.dolcetti@petermac.org (R.D.); pouya.faridi@monash.edu (P.F.)

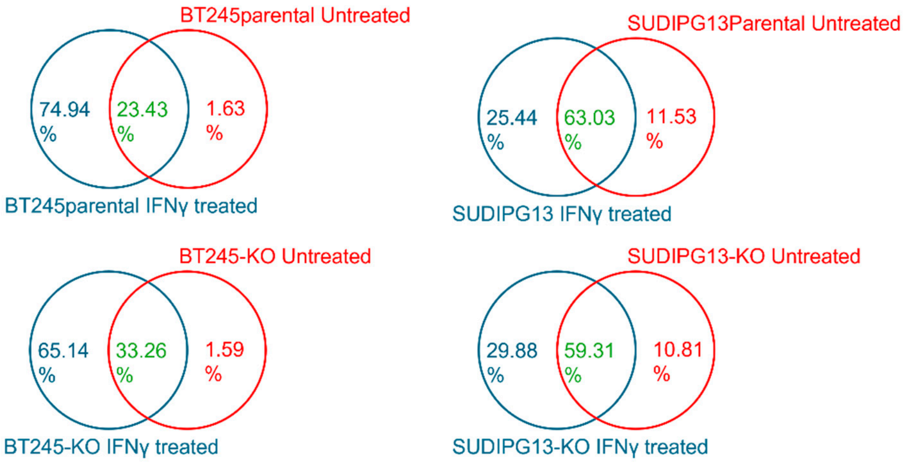


*Supplementary Figure S1. Comparison of pHLA Repertoires in Isogenic H3K27M-DMG Models Before and After IFN-γ Treatment.*

*The overlap of pHLA repertoires between untreated and IFN-γ-treated conditions in BT245 and SU-DIPG13 parental H3K27M-mutant and H3K27M-KO models. Percentages indicate the proportion of peptides unique to each condition (red and blue) and those shared between untreated and IFN-γ-treated samples (green). IFN-γ treatment significantly expanded the shared peptide repertoire, particularly in SU-DIPG13, while in BT245, it covered nearly all peptides present in untreated condition. This highlights the impact of IFN-γ in enhancing antigen presentation.*

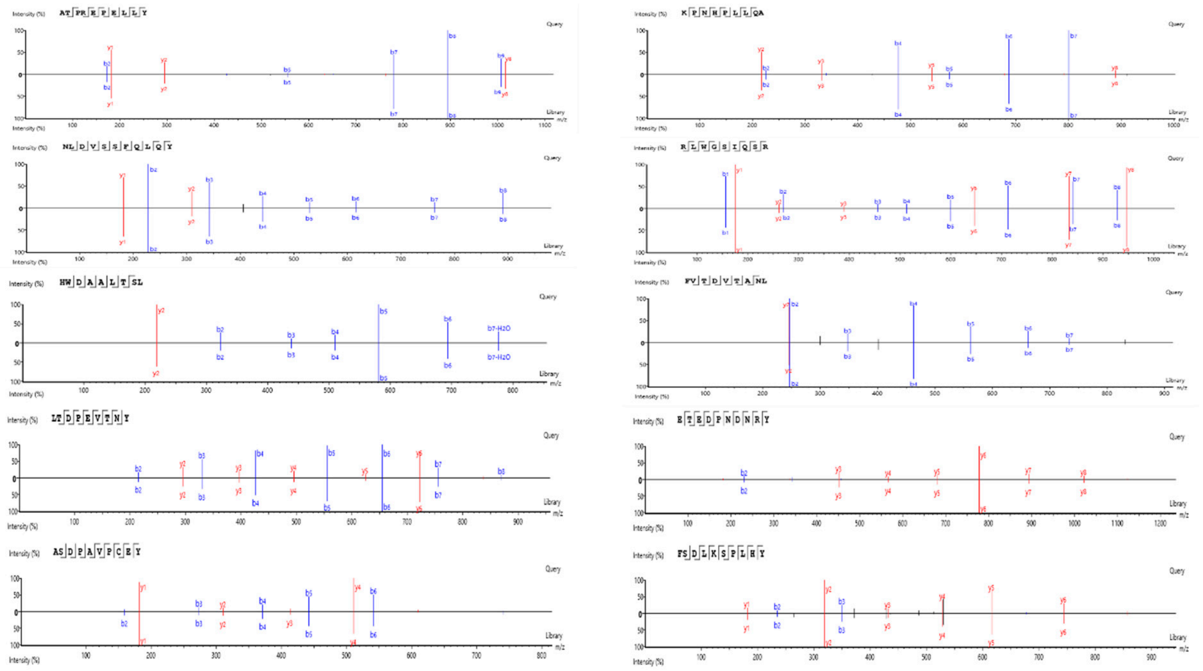

*Supplementary Figure S2. MS Spectra Validation of selected peptides. The actual spectra are shown on the top, while the synthetic spectra are displayed at the bottom (PEAKS 11 software (Bioinformatics Solution Inc).*

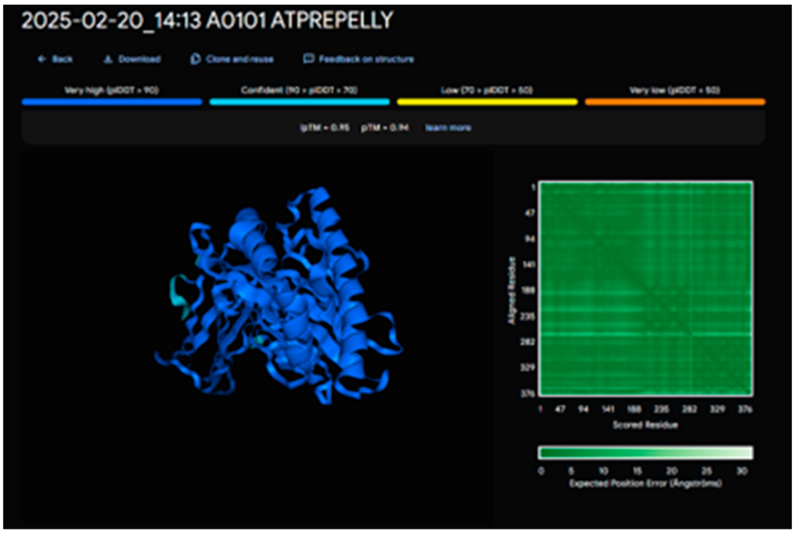
2025-02-20_14:13 A0101 ATPREPELLY
Back
Download
Clone and reuse
Feedback on structure
ipTM = 0.95 pTM = 0.94
learn more
Aligned Residue
Scored Residue
Expected Position Error (Ångströms)

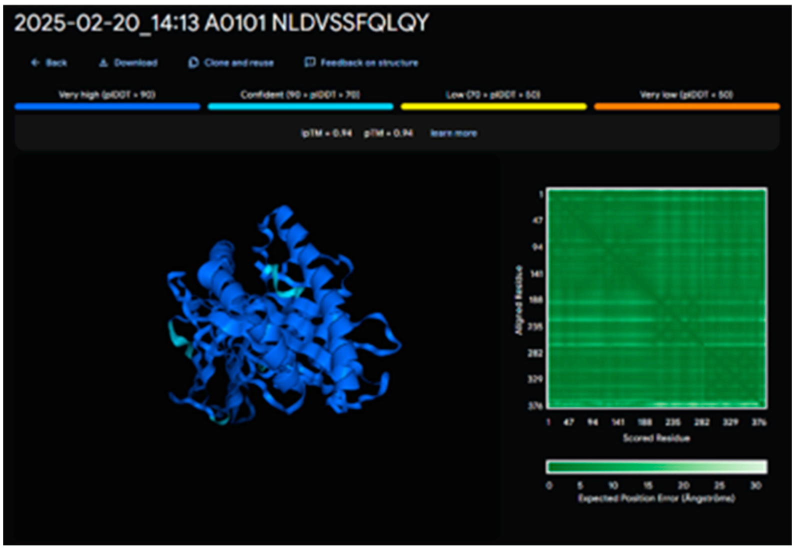
2025-02-20_14:13 A0101 NLDVSSFQLQY
Back
Download
Clone and reuse
Feedback on structure
ipTM = 0.94 pTM = 0.94
learn more
Aligned Residue
Scored Residue
Expected Position Error (Ångströms)

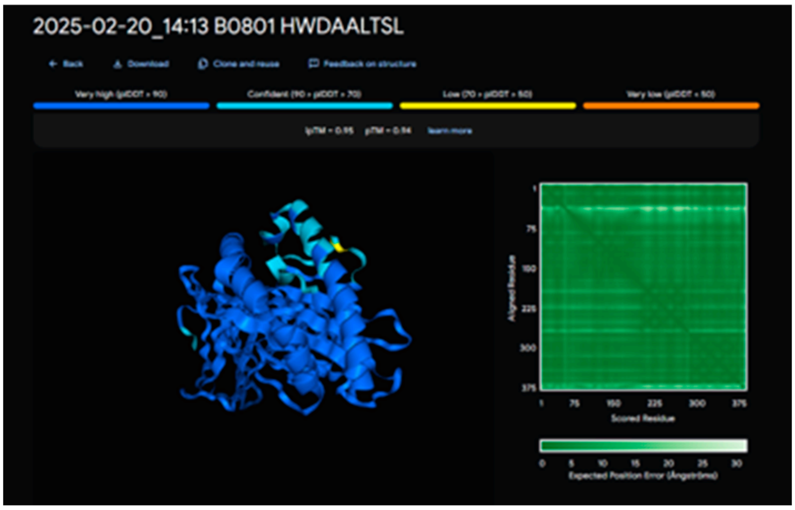
2025-02-20_14:13 B0801 HWDAALTSL
Back
Download
Clone and reuse
Feedback on structure
ipTM = 0.95 pTM = 0.94
learn more
Aligned Residue
Scored Residue
Expected Position Error (Ångströms)

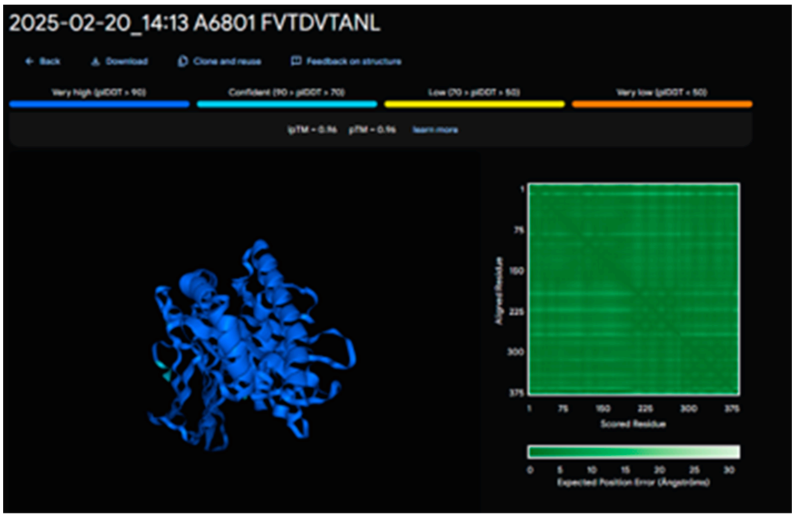
2025-02-20_14:13 A6801 FVTDVTANL
Back
Download
Clone and reuse
Feedback on structure
Very high (plDDT > 90)
Confident (90 > plDDT > 70)
Low (70 > plDDT > 50)
Very low (plDDT < 50)
ipTM = 0.96 pTM = 0.96 learn more
Aligned Residue
Scored Residue
Expected Position Error (Ångströms)

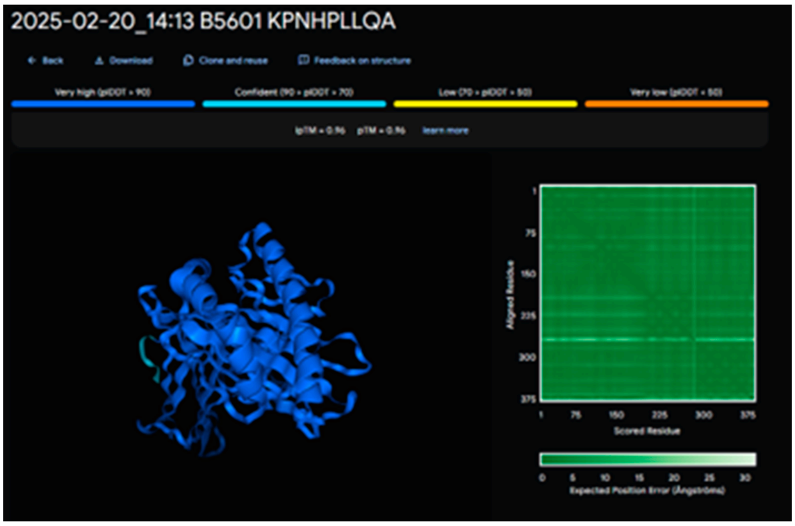
2025-02-20_14:13 B5601 KPNHPLLQA
Back
Download
Clone and reuse
Feedback on structure
Very high (plDDT > 90)
Confident (90 > plDDT > 70)
Low (70 > plDDT > 50)
Very low (plDDT < 50)
ipTM = 0.96 pTM = 0.96 learn more
Aligned Residue
Scored Residue
Expected Position Error (Ångströms)

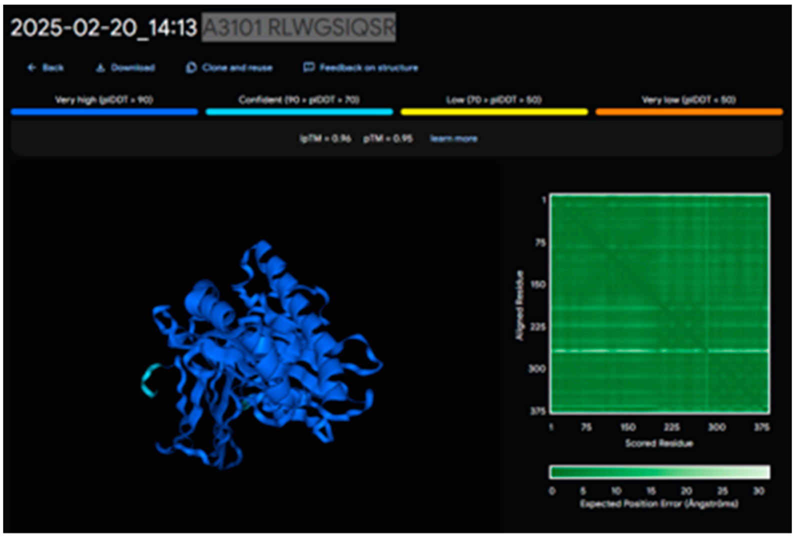
2025-02-20_14:13 A3101 RLWGSIQSR
Back
Download
Clone and reuse
Feedback on structure
Very high (plDDT > 90)
Confident (90 > plDDT > 70)
Low (70 > plDDT > 50)
Very low (plDDT < 50)
ipTM = 0.96 pTM = 0.95 learn more
Aligned Residue
Scored Residue
Expected Position Error (Ångströms)

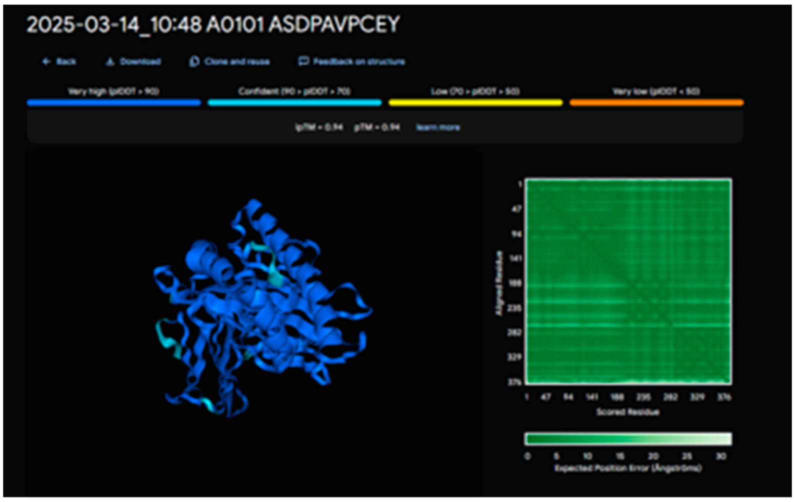
2025-03-14_10:48 A0101 ASDPAVPCEY
ipTM = 0.94 pTM = 0.94

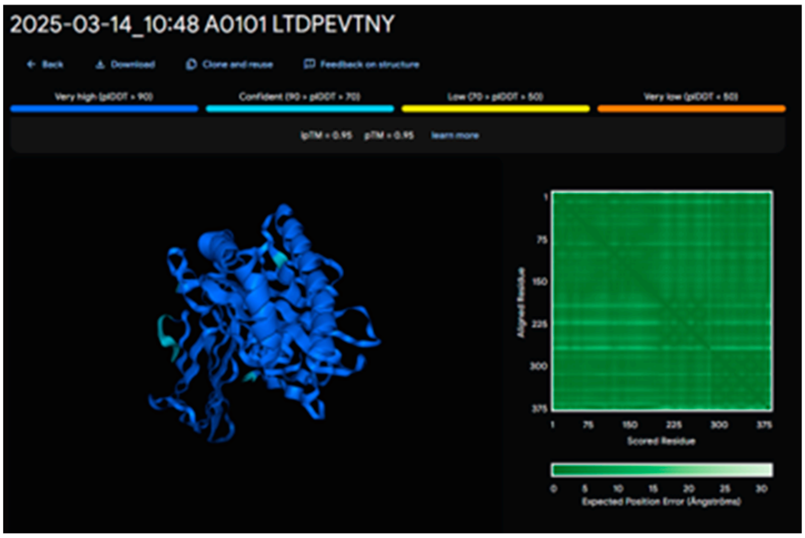
2025-03-14_10:48 A0101 LTDPEVTNY
ipTM = 0.95 pTM = 0.95

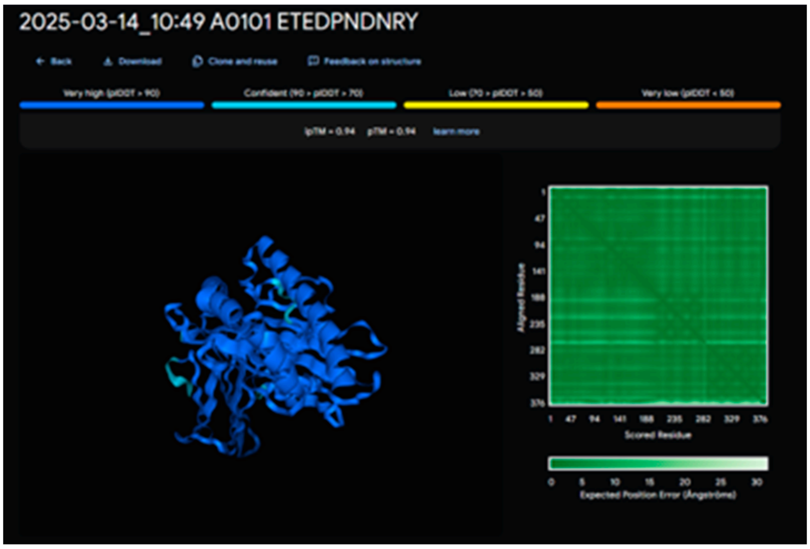
2025-03-14_10:49 A0101 ETEDPNDNRY
ipTM = 0.94 pTM = 0.94

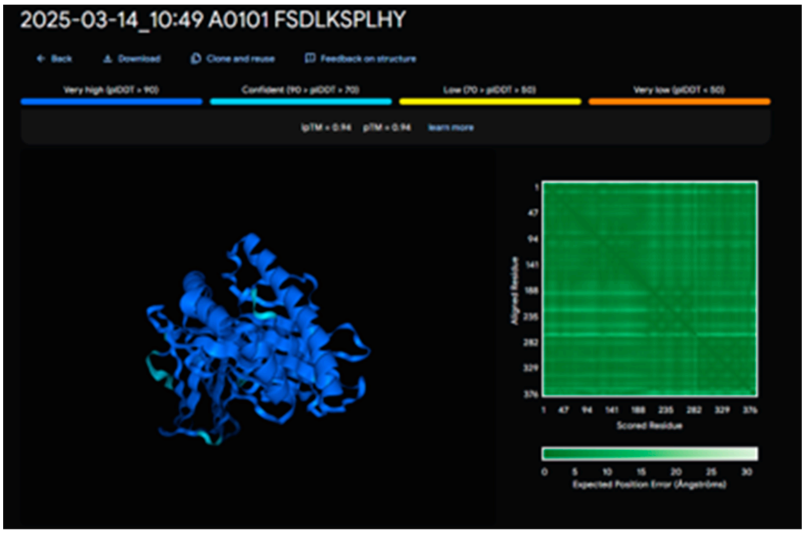


*Supplementary Figure S3. (A–J): Peptides #1–10 prioritised for structural validation and immunogenicity testing. Peptides were selected based on detection in parental H3K27M-mutant cells and non-detection by discovery MS2 in the matched H3K27M-KO models, together with their annotated association with known tumour-associated antigens (TAAs). Left: Predicted 3D structure of the HLA–peptide complex rendered in PyMOL. Right: Structural confidence metrics and modelled peptide placement*

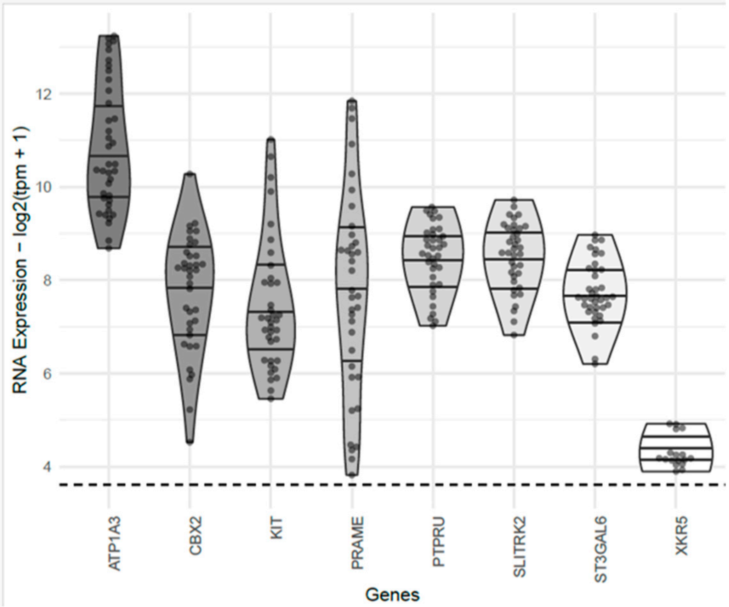


*Supplementary Figure S4. RNA expression of candidate pHLA source genes in DIPG H3K27M autopsy samples. RNA expression values are log2(tpm +1) with variance-stabilising transformation applied from the DESeq2 R package. Expression values for genes in samples for which detected TPM are zero before transformation were not plotted, and a dashed line is shown at this lower limit of detection to aid interpretation.*
